# The role of cholesterol binding in the control of cholesterol by the Scap–Insig system

**DOI:** 10.1007/s00249-022-01606-z

**Published:** 2022-06-19

**Authors:** Anthony G. Lee

**Affiliations:** School of Biological Sciences, University of Southampton, Southampton, SO17 1BJ UK

**Keywords:** Scap, Insig, Molecular docking, Binding energies, Cholesterol, Endoplasmic reticulum

## Abstract

Scap and Insig, two proteins embedded in the membrane of the endoplasmic reticulum (ER), regulate the synthesis of cholesterol in animal cells by forming a dimer in the presence of high concentrations of cholesterol. Cryo-electron microscopic structures for the Scap–Insig dimer show a sterol-binding site at the dimer interface, but none of the structures include cholesterol itself. Here, a molecular docking approach developed to characterise cholesterol binding to the transmembrane (TM) regions of membrane proteins is used to characterise cholesterol binding to sites on the TM surface of the dimer and to the interfacial binding site. Binding of cholesterol is also observed at sites on the extra-membranous luminal domains of Scap, but the properties of these sites suggest that they will be unoccupied in vivo. Comparing the structure of Scap in the dimer with that predicted by AlphaFold for monomeric Scap suggests that dimer formation could result in relocation of TM helix 7 of Scap and of the loop between TM6 and 7, and that this could be the key change on Scap that signals that there is a high concentration of cholesterol in the ER.

**Supplementary Information:**

The online version contains supplementary material available at 10.1007/s00249-022-01606-z.

## Introduction

Central to the control of cholesterol in a mammalian cell is the formation of a dimer, in the endoplasmic reticulum (ER) membrane, between Scap (SREBP cleavage-activating protein, where SREBPs are sterol regulatory element-binding proteins) and Insig (insulin induced gene), dimer formation increasing with increasing cholesterol content of the membrane (Brown et al. 2018). Scap is responsible for the transport and proteolytic activation of SREBP and contains a hexapeptide MELADL sequence in a cytosolic loop that acts as a binding site for vesicle coat protein complex II (COPII). Binding of COPII to a Scap–SREBP complex leads to inclusion into COPII-coated vesicles which bud from the ER and then fuse with the Golgi where SREBPs are cleaved to release their N-terminal transcription factors, these then entering the nucleus to activate many genes involved in the synthesis and uptake of cholesterol (Brown et al. 2018). Formation of the Scap–Insig dimer prevents COPII from binding to the MELADL sequence so that the Scap–SREBP complex remains in the ER (Brown et al. 2018).

**Fig. 1:**
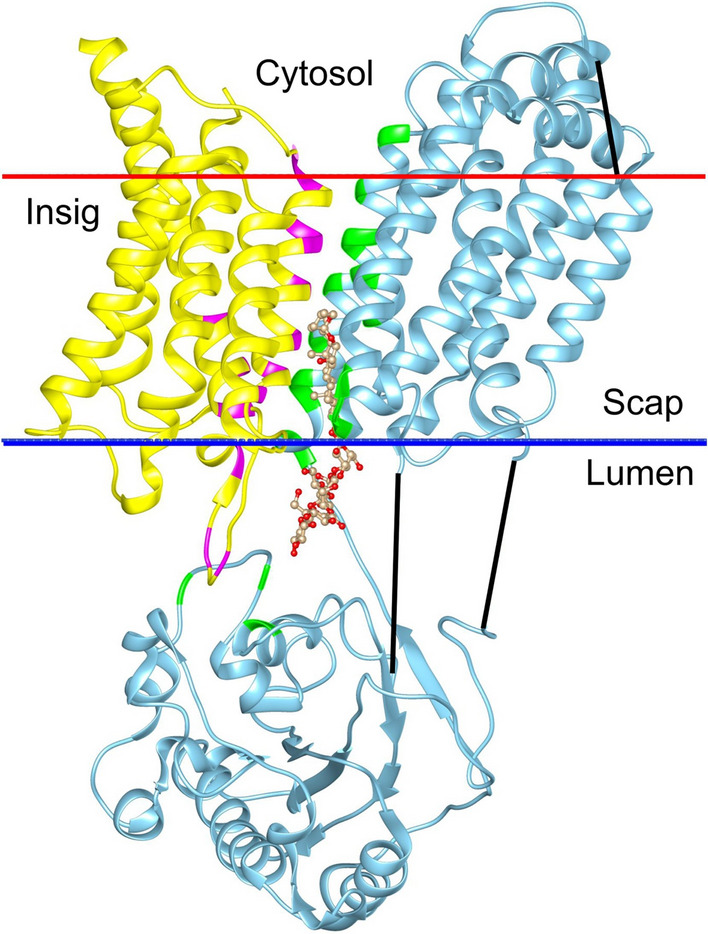
The Scap–Insig dimer (PDB: 7ETW) showing contacts between Scap and Insig. Scap (blue) and Insig (yellow) are shown as ribbon plots. Residues in Insig within 4 Å of a residue in Scap are coloured magenta, and residues in Scap within 4 Å of a residue in Insig are coloured green. The digitonin molecule bound at the dimer interface is shown in ball and stick (*tan*). The black lines show regions of missing structure. The red and blue bars show membrane interfaces on the cytosolic and luminal sides of the ER membrane, respectively, as calculated by the OPM database

The cryo-electron microscopic (cryo-EM) structure (PDB: 7ETW) of the dimer is shown in Fig. 1 (Yan et al. 2021a); this structure is more complete than the other two published structures for the dimer (PDB: 6M49, 7LKF) (Kober et al. 2021; Yan et al. 2021b). Scap and Insig contain eight and six transmembrane (TM) helices respectively. On the cytosolic side, the TM helices of Scap are connected by loops which are short, except for loop L6 between TM6 and 7, which contains ca 94 residues. On the luminal side, loops in Scap are again short, except for loop L1 between TM1 and 2, which contains ca 239 residues, and loop L7 between TM7 and 8, which contains ca 176 residues. In Insig, all TM helices are connected by short loops. Contact between Scap and Insig in the dimer is dominated by the TM helices. There are no contacts between the loops of Scap and Insig on the cytosolic side, although on the luminal side four residues in loop L1 of Insig make contact with three residues in loop L1 of Scap and one residue in loop L7 of Scap (Fig. 1).

**Fig. 2:**
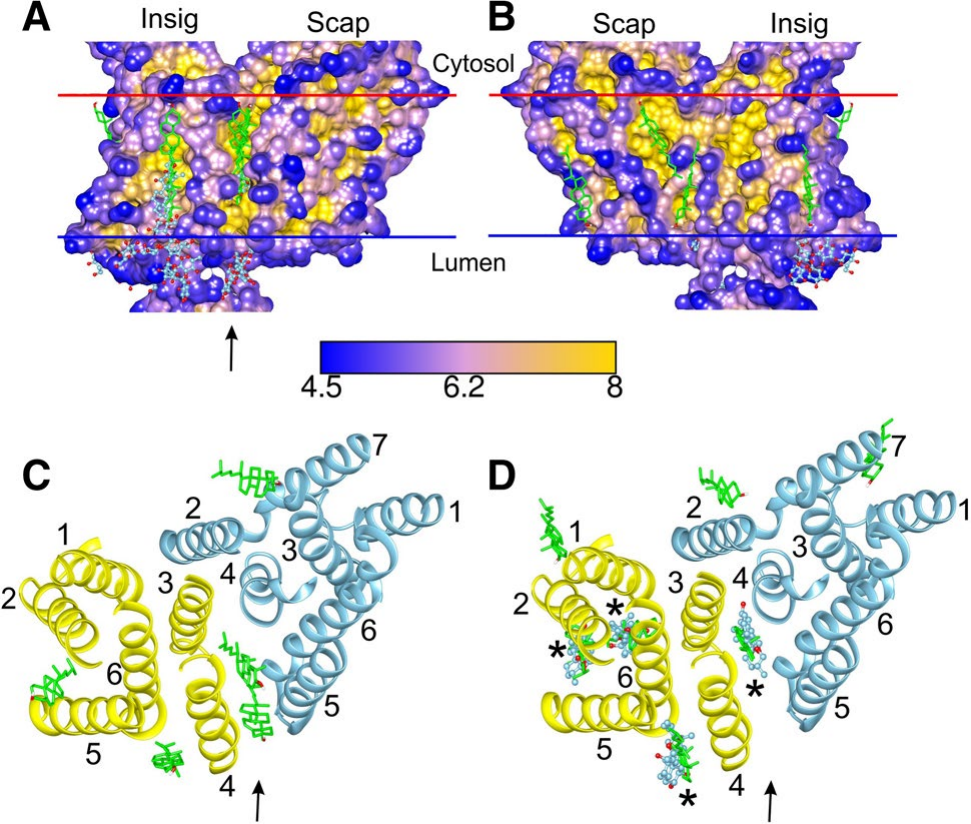
Cholesterol- and digitonin-binding sites on the Scap–Insig dimer. **A** and **B** show surface views of the TM region of the dimer, coloured by depth in Å as given by the scale below. Cholesterol poses are shown in green (sticks) and resolved digitonin are shown in blue (ball and stick). The location of the cleft between Scap and Insig is marked by an arrow in (**A**), and the views in **A** and **B** are related by 180° rotation. **C** and **D** show the TM helices of Scap (blue) and Insig (yellow), both viewed from the cytosolic side, showing molecules bound on the cytosolic (**C**) and luminal (**D**) sides. TM helices are numbered, and the arrows show the position of the cleft between TM3 and 4 of Insig and TM5 and 6 of Scap. The asterisks in (**D**) mark locations where both cholesterol and digitonin are observed; only the sterol ring system of digitonin is shown

Of the 11 residues in the TM region of Insig that make contact with Scap, 7 are hydrophobic, and all of the 16 residues in the TM region of Scap that make contact with Insig are hydrophobic (Fig. 1). Importantly, these hydrophobic interactions will not generate a strong hydrophobic interaction driving dimer formation because the interactions take place in the hydrophobic core of the surrounding phospholipid bilayer; dimer formation will just result in the displacement of hydrophobic fatty acyl chains by hydrophobic amino acid residues. Dimer formation does, however, depend on good packing between the TM surfaces of Scap and Insig, and, in membrane proteins, these surfaces are generally rough, consisting of a series of ridges and hollows (Lee 2019a, b, 2020, 2021), as shown in depth plots (Tan et al. 2013) for the dimer (Fig. 2A, B). Dimer formation would be enhanced if molecules of cholesterol or phospholipid present in the ER membrane could fill any gaps at the dimer interface. The abilities of these molecules to fill any such gaps are likely to differ because of differences in their flexibilities. The limited data available suggest that binding sites for phospholipids are more likely to be on ridges than in hollows, because their flexible fatty acyl chains are able to distort to match the rough surfaces of the ridges, whereas cholesterol molecules are more likely to be found in the hollows because of the limited ability of the more rigid cholesterol molecule to distort (Lee 2019b, 2020).

No high-resolution structures are yet available for a Scap–Insig dimer with bound cholesterols, but structures are available with 25-hydroxycholesterol (25-HC) or the steroidal detergent digitonin bound at the dimer interface, the latter structure also showing three additional digitonins bound between the TM helices of Insig, in surface hollows (Fig. 2) (Yan et al. 2021a, b). Binding studies suggest that cholesterol can also bind to constructs corresponding to loop L1 of Scap and to a complex between loops L1 and L7 (Motamed et al. 2011; Zhang et al. 2016; Brown et al. 2018), but no bound sterol molecules have been resolved in the loop regions in cryo-EM studies (Kober et al. 2021; Yan et al. 2021a, b).

Understanding the pathway for dimer formation requires a knowledge of the structures of monomeric Scap and Insig as well as that of the dimer. An X-ray structure is available for a bacterial homolog of Insig (Ren et al. 2015), which adopts a structure very similar to that adopted by Insig in the Scap–Insig dimer. Unfortunately, attempts to obtain a high-resolution structure for monomeric Scap have not been successful, but a partial structure suggests that the packing of TM helices in the monomer and the Scap–Insig dimer are different (Kober et al. 2021). It will be suggested below that the structure for the Scap monomer predicted by AlphaFold (Jumper et al. 2021) could correspond to the structure of the cholesterol-free Scap monomer.

It is clear that new approaches will be necessary if we are to understand how cholesterol drives Scap–Insig dimer formation. In previous publications, it has been shown that a molecular docking approach can be used to study the binding of cholesterol to the TM regions of G-protein coupled receptors (GPCRs), potassium channels, TRP ion channels, and GABA_A_ receptors, providing information complementary to that provided by X-ray and cryo-EM studies (Lee 2019a, b, 2020, 2021). These studies have shown that molecular docking can often be more successful than molecular dynamic (MD) simulations in identifying cholesterol-binding sites. The high concentrations of cholesterol in a biological membrane mean that a large fraction of the cholesterol molecules on the TM surface of a membrane protein at any given time will be there as a result of random collisions rather than as a result of binding at some particular ‘hot spot’, and distinguishing between these possibilities is not easy in MD simulations, although this would be helped using binding affinities which can now be derived from the simulations (Ansell et al. 2021; Lee and Lyman 2012). Further, the mobility of a cholesterol molecule in its binding site makes it difficult to identify the protein residues contributing to a particular binding site, particularly if these sites are close together (Rouviere et al. 2017; Barbera et al. 2018; Duncan et al. 2020; Sejdiu and Tieleman 2020) although recently developed procedures, such as ProLint (Sejdiu and Tieleman 2021) and PyLipID (Song et al. 2022), make this more readily achievable. In X-ray crystallography and cryo-EM these problems are solved by freezing out molecular motion by working at low temperatures, and molecular docking, by working with these rigid protein structures, can, in suitable cases, give ligand binding poses that correspond to binding sites on the X-ray crystallography and cryo-EM structures. Of course, this is at the cost of the valuable information about dynamics which is provided by MD approaches.

A potential limitation of the docking approach is that it can find it difficult to identify strong binding sites of the type characteristic of drug binding, because of the presence of specific, directional interactions between drug and protein. However, a docking approach works well with cholesterol-binding sites on the TM surfaces of membrane proteins because these sites are structurally promiscuous and relatively weak, with few, if any, specific interactions; the hollows where cholesterol molecules bind are not deep energy wells into which a cholesterol molecule falls, adopting a single pose, but are shallow energy wells able to accommodate cholesterol molecules in a variety of poses (Rouviere et al. 2017; Barbera et al. 2018; Hedger et al. 2019; Lee 2019a, b, 2020, 2021). Despite the relatively weak binding at these sites, occupancy of the sites by cholesterol will be high because the mole fractions of cholesterol in the plasma and ER membranes are high, ca 0.3 and 0.1, respectively (Song et al. 2014; van Meer et al. 2008). A similar promiscuity in binding exists at the interfacial dimer site in the Scap–Insig dimer, as demonstrated by comparing the binding poses adopted by digitonin and 25-HC (Yan et al. 2021a, b) (Fig. 1 of supplementary information). Although both clearly bind in the same location, and although both binding sites are composed of ten residues, both with six residues from Scap and four from Insig, only four of these residues are common to the two binding sites (Fig. 1 of supplementary information). These differences are perhaps not surprising given that the steroid ring of digitonin, unlike that of cholesterol, contains two –OH groups and 2 ring oxygens.

The approach used here employs the AutoDock Vina molecular docking programme (Trott and Olson 2010) to sweep the TM surface of a membrane protein for cholesterol-binding sites. An analysis of cholesterol molecules bound to TM sites shows that cholesterol –OH groups are generally located close to one of the two polar–hydrophobic interfaces of the surrounding lipid bilayer and that they are more likely to form hydrogen bonds with a bilayer interface than with the protein (Rouviere et al. 2017; Barbera et al. 2018; Hedger et al. 2019; Lee 2019a, b, 2020, 2021). The docking procedure therefore includes, as well as the membrane protein, layers of hydrogen bond donors and acceptors, located at the positions of the two membrane interfaces as calculated by the Orientations of Proteins in Membrane (OPM) database (Lomize et al. 2006). Docking results in a large number of possible cholesterol poses that have to be sorted to separate poses corresponding to binding sites of the type seen in X-ray and cryo-EM studies (‘hot-spots’) from weak, background sites and from false sites; in any docking study it is important to minimise the number of false sites, even at the expense of missing some true sites (Kitchen et al. 2004). Sorting is achieved using an evidence-based approach, selecting just those poses that match the characteristics of known cholesterol-binding sites on membrane proteins, defined from studies of the large numbers of bound cholesterol molecules resolved in crystallographic studies of GPCRs (Lee 2019a); the selection criteria are that a cholesterol-binding site should contain a minimum of eight residues within 4 Å of the bound cholesterol and that the angle between the long axis of the cholesterol ring system and the bilayer normal should be less than 30°. Docking to the TM domains uses binding parameters appropriate for a hydrophobic environment (Lee 2019a). Docking studies were also carried out for the extra-membranous domains of Scap and these used the default binding parameters of AutoDock Vina, derived for studies of binding of ligands in an aqueous environment (Trott and Olson 2010).

In this study, it will be shown that molecular docking can be used to characterise cholesterol binding to the TM and extra-membranous domains of Scap, of Insig, and of the Scap–Insig dimer. It will be shown that the cholesterol-binding site at the dimer interface is a shallow energy well capable of accommodating a wide variety of sterols, consistent with in vivo studies of the effects of sterol structure on retention of Scap in the ER membrane (Radhakrishnan et al. 2007). Although the docking studies also suggest the presence of potential binding sites for cholesterol on the luminal domains of Scap, it will be argued that the characteristics of these sites are such that they unlikely to be occupied in vivo, suggesting that formation of the Scap–Insig dimer can be understood solely in terms of cholesterol binding to its site at the dimer interface. It will also be suggested that the Scap structure predicted by AlphaFold (Jumper et al. 2021) could correspond to that of the cholesterol-free monomer and that binding of Scap to Insig to form a dimer involves a relocation of TM7 of Scap. This, in turn, would result in a change in the conformation of the loop between TM6 and 7, occluding the MELADL sequence in the loop and blocking binding of COPII-coated vesicles so that the Scap–Insig dimer with its bound SREBP remains in the ER.

Details of all the docking results are available in the Supplementary Information and are available for downloading as PDB files on the DeepCholesterol web site (https://deepcholesterol.soton.ac.uk).

## Methods

The docking protocol used has been described in detail elsewhere (Lee 2021). Structures were downloaded from the OPM database (http://opm.phar.umich.edu) (Lomize et al. 2006) and the dummy atoms marking the membrane interfaces in these structures were converted into NH_3_ groups using in-house Python code. Docking was performed using AutoDock Vina (Trott and Olson 2010) running under Chimera (Pettersen et al. 2004). Proteins were prepared for docking using the DockPrep and AddH routines in Chimera and incomplete side chains were repaired using the Dunbrack rotamer library (Pettersen et al. 2004); any ligands were deleted before docking. Cholesterol was prepared for docking with free rotation about the C–OH bond, using AutoDock 4 (Morris et al. 2009). Docking to the TM domain used weighting factors of – 0.001 and – 2.0 for the hydrophobic effect and for hydrogen bonding, respectively (Lee 2019a) and docking was performed separately for the cytosolic and luminal halves of the membrane, with two overlapping search boxes for each half; duplicate docking poses in the regions of overlap of the boxes were removed. Five sequential docking runs were performed for each search box and binding sites of the type seen in X-ray and cryo-EM studies were separated from weak, background and “false” sites by selecting just those poses that matched the characteristics of known interfacial cholesterol-binding sites (Lee 2019a); the selection criteria were that a binding site should contain a minimum of eight protein residues within 4 Å of a bound cholesterol and that the angle between the long axis of the cholesterol ring system and the bilayer normal should be less than 30°. Poses were sorted into clusters using simple threshold clustering (Stevens and Boucher 2015) based on a root mean square deviation (rmsd) of less than 4 Å and the energetically most favourable pose in a cluster was chosen to represent that cluster. The stochastic approach used by Vina includes random factors that do not allow full reproducibility (Yuriev et al. 2015) and this is most obvious when docking to large structures such as the Scap–Insig dimer, not in the actual sites detected but, for a small number of sites, in whether or not the site is occupied. Reproducibility was therefore improved by performing five independent docking studies for each structure, followed by clustering of the selected cholesterol poses from the five studies; only poses from clusters containing a member from each of the five studies were accepted (Lee 2021). Docking to the luminal domains of Scap used the default weighting factors of – 0.0351 and – 0.587 for the hydrophobic effect and for hydrogen bonding, respectively (Trott and Olson 2010), without the selection criteria used for docking in the TM region.

Residues within 4 Å of a docked cholesterol were identified using Chimera (Pettersen et al. 2004). Surface depths were obtained using the Depth server (Tan et al. 2013) with a solvent neighbour radius of 3 Å, a minimum number of neighbourhood solvent molecules of 5 and a number of solvating cycles of 100. Sterol structures were downloaded from the PubChem database (https://pubchem.ncbi.nlm.nih.gov/). AlphaFold structures were downloaded from the ebi database (https://alphafold.ebi.ac.uk) (Jumper et al. 2021; Varadi et al. 2022).

## Results and discussion

### Validation of the docking approach for TM sites

A test of the validity of any docking approach is its ability to reproduce known binding sites for the ligand of interest. Figure 2 shows the TM region of the Scap–Insig dimer determined in the presence of the steroidal detergent digitonin (Yan et al. 2021a). No digitonin are resolved on the cytosolic side of the membrane but four digitonin are resolved on the luminal side, one at the Scap–Insig dimer interface, the other three being bound on the TM surface of Insig (Fig. 2D). Importantly, all four of these resolved digitonin are matched by cholesterol poses, arguing both that the digitonin-binding sites are likely to represent binding sites for cholesterol and providing confidence in the docking approach used here. Digitonin molecules have also been resolved on the SUR subunit of the potassium channel Kir6.2 (Ding et al. 2019), and, in unpublished studies, have also been shown to be matched by cholesterol poses. As shown in Fig. 1 of the supplementary information, 25-HC occupies the same site as digitonin at the dimer interface.

### Cholesterol binding to the Scap–Insig dimer

Depth plots for the Scap–Insig dimer show that bound digitonin and cholesterol molecules are all located in hollows in the surface or in the cleft at the Scap–Insig interface (Fig. 2A, B). Cholesterol poses are located with their –OH groups close to the two membrane interfaces, as calculated by OPM (Lomize et al. 2006), and digitonins are located similarly with the sugar-linked O atom of the steroid group, equivalent to the –OH group of cholesterol, also being close to an interface. The cholesterol and digitonin molecules show extensive hydrogen bonding to the interface, but none to the protein.

Cholesterol poses are observed at sites where digitonin molecules are not resolved as well as at sites where they are (Fig. 2). It is, of course, not possible to say whether or not these additional poses are “real”, a problem faced by any in silico approach. However, all the cholesterol poses are located in surface hollows and all were selected on the basis that they showed the properties characteristic of cholesterol molecules resolved on a wide variety of other membrane proteins (Lee 2019a, b, 2020, 2021). Further, the average docking energy of the poses, – 14.2 ± 1.1 kcals mol^−1^ (Table 3 of supplementary information), is very similar, for example, to that for the GABA_A_ receptor, which is – 13.5 ± 0.7 kcals mol^−1^ (Lee 2021). It is possible that digitonin is prevented from binding to some cholesterol-binding sites by its large sugar groups. More generally, the loose nature of the binding sites could mean that, at some sites, bound digitonin molecules are too mobile to be resolved; it is also possible that the overall resolution of the structure is too low to allow resolution of all the bound digitonin molecules.

**Fig. 3:**
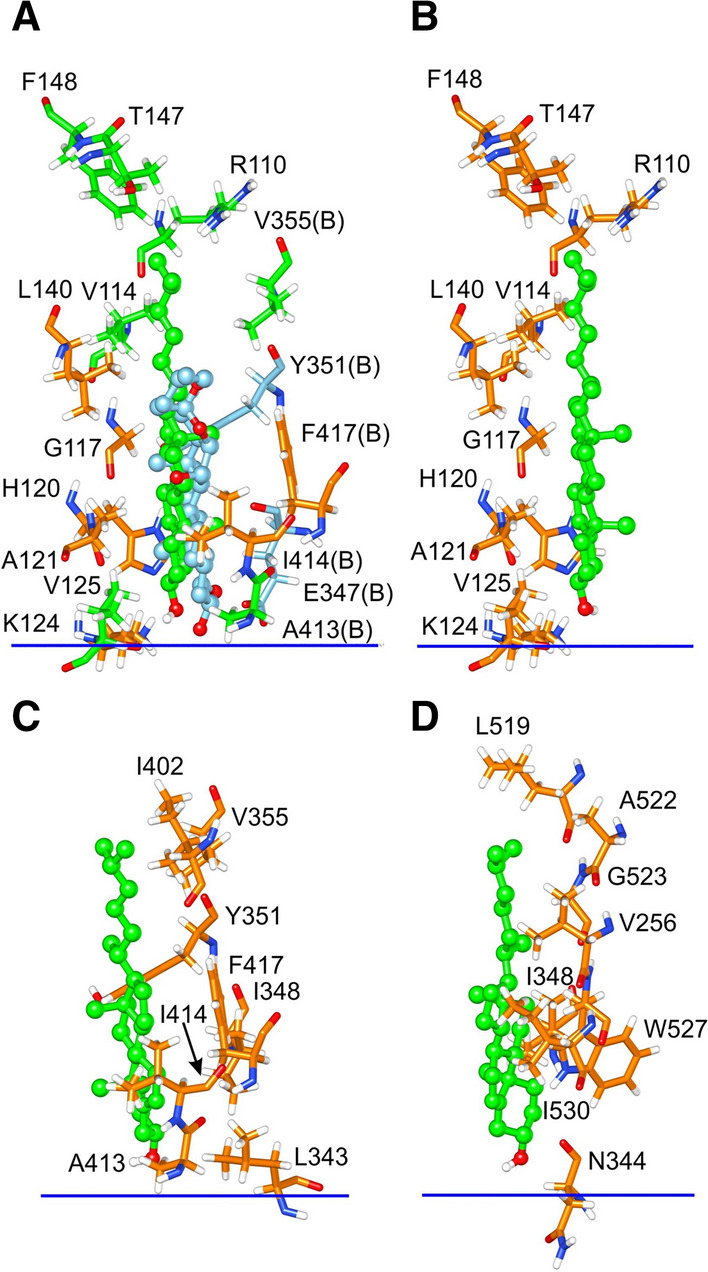
Cholesterol binding to the sterol binding site at the Scap–Insig dimer interface and at equivalent sites on monomeric Scap and Insig. Bound cholesterol (green) are shown as ball and stick. Residues within 4 Å of the bound cholesterol are shown as sticks. **A** shows binding of cholesterol at the interfacial site on the Scap–Insig dimer together with the digitonin molecule (blue, ball and stick) resolved at the dimer interface; for simplicity only the sterol ring system of digitonin is shown. Residues close to both cholesterol and digitonin are coloured orange, those close to just cholesterol are green, and those close to just digitonin are blue. All residues are from the Insig subunit unless marked B, which are from Scap. In (**B–D**) all local residues are coloured orange. **B** and **C** show the binding sites equivalent to the interfacial site shown in (**A**)*,* on monomeric Insig and Scap respectively, in their dimer conformations. **D** shows the cholesterol-binding site closest to the interfacial site shown in (**A**), for the AlphaFold model for monomeric Scap (Jumper et al. 2021). The blue bars show the interface on the luminal side of the ER membrane

The large cleft at the interface between Scap and Insig extends from one side of the membrane to the other, between TM3 and 4 of Insig and TM5 and 6 of Scap (Fig. 2). On the luminal side the cleft can be occupied by digitonin or 25-HC (Yan et al. 2021a, b) (Fig. 1 of supplementary information), and is the site of a cholesterol pose (Fig. 2D). Although no bound digitonin were resolved on the cytosolic side of the cleft, this is the location for two cholesterol poses (Fig. 2C). The innermost of these cholesterols makes contact with 9 residues in Insig and 5 in Scap and has a docking energy of – 14.7 kcals mol^−1^; the outermost cholesterol contacts 6 residues in Insig and 4 residues in Scap, and has a docking energy of – 14.3 kcals mol^−1^. These docking energies are less than that for the single cholesterol located on the luminal side of the cleft (– 16.8 kcals mol^−1^; Table 1). The cleft is open to the aqueous medium on both the cytosolic and luminal sides and is also open to the hydrophobic core of the surrounding lipid bilayer along most of its length, although it is closed off from the lipid bilayer along a short length on the luminal side by Gln-132 in Insig and Thr-409 and Val-411 in Scap (Fig. 2A). It is likely therefore that a phospholipid molecule will be able to bind on the cytosolic side with one or both of its fatty acyl chains in the cleft but that this will not be possible on the luminal side; binding of phospholipids and cholesterol in clefts has been shown to be competitive for a number of membrane proteins (Lee 2019b). Weak binding of cholesterol on the cytosolic side of the cleft, combined with competition with phospholipids for binding, means that cholesterol is more likely to be bound on the luminal side of the cleft than on the cytosolic side. In what follows, the discussion will therefore concentrate on the luminal site, which will be referred to simply as the dimer site. Binding of cholesterol and digitonin at the dimer site are compared in Fig. 3A. The sterol rings occupy very similar positions in the site despite the fact that the digitonin steroid ring, as well as its sugar-linked oxygen atom, contains two –OH groups and 2 ring oxygens, illustrating the structural promiscuity of the site, consistent with the wide range of sterols that have been shown to be able to trap Scap in the ER membrane (Radhakrishnan et al. 2007).

**Table 1:** Docking and binding energies (kcals mol^−1^) for cholesterol at the interfacial site on the Scap–Insig dimer and at the equivalent sites on Scap and Insig monomers

Protein	Docking energy		Binding parameters (mole fraction units)	
	Molar units	Mole fraction units	Binding energy corrected for H bonding to bilayer	*K*_a_
Scap–Insig dimer	– 16.8	– 17.5	– 7.1	1.6 × 10^5^
Insig monomer^a^	– 14.0	– 14.7	– 4.3	1.4 × 10^3^
Scap monomer^a^	– 12.5	– 13.2	– 2.8	1.1 × 10^2^
Scap monomer^b^	No Binding			

^a^With the structure adopted in the Scap–Insig dimer

^b^AlphaFold structure for the cholesterol-free Scap monomer

The docking energy for cholesterol at the dimer site on the Scap–Insig dimer is given in Table 1. In a standard docking procedure, the energy of interaction is calculated between a bare ligand molecule and a bare protein molecule. However, here a bare cholesterol molecule is docked to a bare protein molecule surrounded by two bare bilayer interfaces, all in a hydrophobic environment, so that docking energies will include hydrogen bonding between cholesterol and protein and between cholesterol and the interfacial region of the lipid bilayer. It has been suggested that a multiplex hydrogen bond in which a single hydrogen bond acceptor interacts with multiple hydrogen bond donors is ca 60% stronger than a single, canonical hydrogen bond (Feldblum and Arkin 2014; Brielle and Arkin 2020) and the hydrogen bond detector in Chimera (Pettersen et al. 2004) suggests that multiplex hydrogen bonds are formed between a cholesterol –OH and the bilayer interface. Concentrations of cholesterol in a membrane are best expressed in mole fraction units and the standard free energy for formation of a canonical hydrogen bond in a hydrophobic environment in mole fraction units is – 6.5 kcals mol^−1^ (Ben-Tal et al. 1997; Feldblum and Arkin 2014) giving an energy for a multiplex hydrogen bond between cholesterol and the bilayer interface of ca – 10.4 kcals mol^−1^; this was the value used to interpret binding energies for cholesterol with GABA_A_ receptors (Lee 2021). The standard free energy Δ*G*^o^ of binding for cholesterol at the dimer site on the Scap–Insig dimer is – 17.5 kcals mol^−1^ in mole fraction units (Table 1); this value includes both hydrogen bonding to the bilayer interface and to the protein. Subtracting the term for hydrogen bonding to the interface gives a value for Δ*G*^o^ for binding to the protein alone of – 7.1 kcals mol^−1^ corresponding to an association constants *K*_a_ (Δ*G*^o^ = – *RTlnK*_*a*_) of 1.6 × 10^5^ (Table 1). The likely reliability of estimates of Δ*G*^o^ derived from docking studies is discussed in the Supplementary Information, based on a comparison with binding energies obtained from MD simulations on GPCRs (Tables A1 and A2 of supplementary information).

The estimated value for *K*_a_ means that the dimer site will be 50% occupied at a cholesterol concentration of 0.0006 mol%, which compares with a cholesterol concentration of 5.5 mol% for half maximal SREBP processing at normal Insig levels (Radhakrishnan et al. 2008). These very large differences in concentration raise the question of whether or not the cholesterol-binding site at the Scap–Insig dimer interface could be the site controlling the cholesterol dependence of SREBP processing, but it is shown in the Supplementary Information that this is not a problem. When considering the cholesterol dependence of SREBP processing it is necessary to consider not only cholesterol binding to the Scap–Insig dimer but also the formation of the Scap–Insig dimer from monomeric Scap and Insig in the membrane. As shown in the Supplementary Information, the proportion of Scap and Insig in the membrane that is present as a dimer depends on the concentrations of Scap and Insig in the membrane. Although these are not known, we do know that they will be low (in mole fraction units) because of the high molar ratios of lipid to protein in biological membranes such as the ER. For example, the molar ratio of lipid to protein in the membrane of the sarcoplasmic reticulum is ca 100:1, or 50:1 in each monolayer (Lee 2003). If then, for example, Scap and Insig each make up 5% of the protein molecules in the membrane, their mole fractions will be 0.001 and these low values will favour monomeric Scap and Insig over the Scap–Insig dimer. It is shown in the Supplementary Information that an association constant for Scap and Insig of 4, combined with the cholesterol binding constant for the dimer of 1.6 × 10^5^ (Table 1), means that with Scap and Insig each making up 5% of the protein molecules in the membrane, the cholesterol concentration required for 50% of the Scap and Insig molecules to be present as the cholesterol-bound Scap–Insig dimer, will be 5.5 mol %, matching the value determined by Radhakrishnan et al. (2008) for half maximal SREBP processing; matching at other values for the mole fractions of Scap or Insig simply requires changing the association constant for Scap and Insig (see Supplementary Information). Radhakrishnan et al. (2008) found that the effect of cholesterol on SREBP processing was co-operative; although binding of cholesterol to a single site on the Scap–Insig dimer will show no cooperativity, the transport of SREBP bound to Scap, from the ER membrane to the Golgi apparatus in COPII-coated vesicles, will be co-operative, as packing of membrane proteins into COPII-coated vesicles has been shown to depend on the oligomeric state of the protein (Springer et al. 2014). Radhakrishnan et al. (2008) also found that the concentration of cholesterol in the ER membrane required for half maximal SREBP processing decreased from ca 5.5 mol% at normal levels of Insig to 3.1 mol% when the level of Insig was increased, also consistent with the analysis presented here (see Supplementary Information). It is concluded therefore that the cholesterol-binding site at the Scap–Insig dimer interface could be the site controlling the cholesterol dependence of SREBP processing, and that it is not necessary to invoke a role for any other binding sites for cholesterol on the Scap–Insig dimer.

### Cholesterol binding to Scap and Insig monomers

**Fig. 4:**
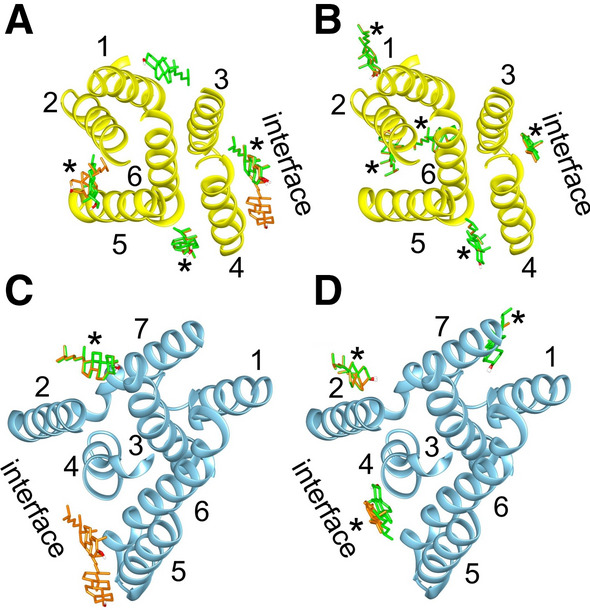
Cholesterol binding to monomeric Scap and Insig in their dimer conformations. All views are from the cytosolic side with the TM helices shown as ribbons. **A** and **B** show binding to Insig and **C** and **D** show binding to Scap, with **A** and **C** showing binding on the cytosolic side and **B** and **D** showing binding on the luminal side. Cholesterol bound to monomeric Scap and Insig are shown in green and cholesterol bound to Scap and Insig in the dimer are shown in orange; results for the dimer are from Fig. 2. The asterisks mark sites occupied in both the monomeric and dimeric structures, and the position where the dimer interface would be in the Scap–Insig dimer is marked

Scap and Insig will exist in the ER membrane as monomers as well as dimers. Generally comparable patterns of cholesterol docking are observed for the Scap–Insig dimer and for the component monomers in the conformations they adopt in the dimer (Fig. 4). Importantly, binding of cholesterol is seen at sites on the luminal sides of monomeric Scap and Insig equivalent to the dimer site (Fig. 4B, D); these sites will be referred to as the Scap dimer site and Insig dimer site, respectively. Of the two binding sites for cholesterol on the cytosolic side of the interfacial cleft in the dimer (Fig. 2C), only one is seen on the Insig monomer and neither are seen on the Scap monomer (Fig. 4A, C). An additional binding site is seen on the Insig monomer on the cytosolic side between TM1 and 3 (Fig. 4A), a region occupied by TM2 of Scap in the dimer. Residues making up the Insig and Scap dimer sites on the monomers are shown in Fig. 3B, C; those on the Insig monomer (Fig. 3B) are identical to those contributed by the Insig monomer to the binding site on the Scap–Insig dimer (Fig. 3A), and those on the Scap monomer (Fig. 3C) include five of the six residues contributed by the Scap monomer to the binding site on the Scap–Insig dimer (Fig. 3A).

Docking energies at the Scap and Insig dimer sites on the monomers are given in Table 1, together with the estimated binding energies. Although converting from docking energies to binding energies requires a number of assumptions, as described above, it is interesting that the binding energy at the dimer site on the Scap–Insig dimer is equal to the sum of the binding energies at the Scap and Insig dimer sites (Table 1). This implies that the contributions of Scap and Insig residues to dimer formation are independent, with Insig making the greatest contribution; as described by Yan et al. (2021a, b), the cholesterol molecule acts as a simple molecular glue, holding together the two sides of the binding site.

Unfortunately, it is not possible to compare these docking results with the results of direct binding studies with cholesterol, made using a construct consisting of TM1-8 of Scap expressed and purified from insect cells (Radhakrishnan et al. 2004, 2007). These studies showed saturable binding of cholesterol, but with a maximum binding of either 1 cholesterol per 62 Scap molecules (Radhakrishnan et al. 2004) or 1 cholesterol per 30 Scap molecules (Radhakrishnan et al. 2007). In contrast, the docking studies with Scap in its dimer conformation (Fig. 4) suggested 4 cholesterol-binding sites per Scap molecule. It is not obvious how the direct binding results should be mapped onto the docking results. The binding experiments made use of the detergent Fos-choline 13, and Radhakrishnan et al. (2007) suggested that the low binding stoichiometry could have been due to an effect of Fos-choline 13 on the structure of the construct; the Fos-cholines are harsh detergents that denature many α-helical membrane proteins (Chipot et al. 2018; Kotov et al. 2019). It is also possible that protein-bound cholesterol could have been lost during the ca. 20 min wash procedure with detergent-containing buffer, used to separate bound and non-bound cholesterol. Unfortunately, it is also not possible to compare binding constants from these studies with those from docking, because, as discussed by Radhakrishnan et al. (2004), the binding studies correspond to a detergent micelle environment whereas the docking studies were designed to represent a lipid bilayer environment. Direct binding experiments with 25-HC failed to detect any saturable binding (Radhakrishnan et al. 2004), possibly because the higher polarity of 25-HC than of cholesterol led to a faster wash-off rate.

Direct binding studies with Insig-2 gave maximal binding levels per Insig molecule for cholesterol and 25-HC of 1 per 266 and 1 per 73, respectively (Radhakrishnan et al. 2007); again, it is not obvious how these results should be compared to the docking or cryo-EM studies; docking studies with monomeric Insig suggest nine binding sites for cholesterol (Fig. 4) per monomer and the cryo-EM structure (PDB: 7ETW) shows four binding sites for digitonin on the Insig subunit in the Scap–Insig dimer (Fig. 2).

### AlphaFold models for monomeric Scap and Insig

**Fig. 5:**
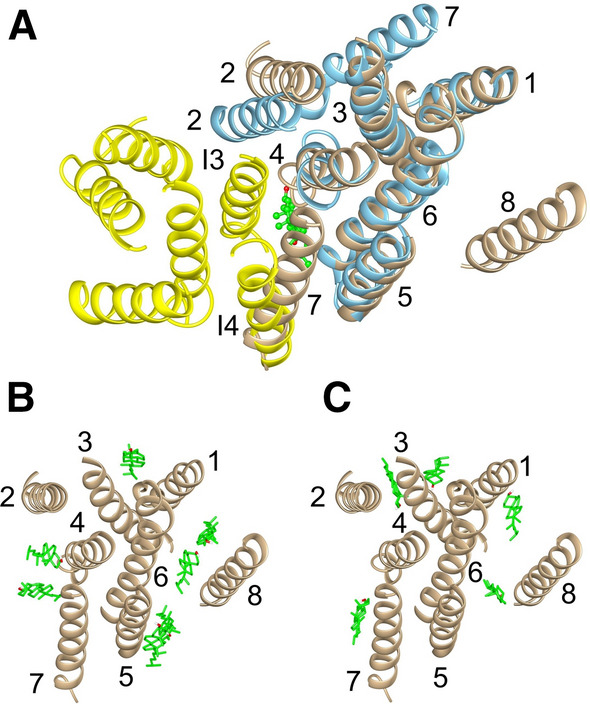
Comparison of the TM region of the AlphaFold model for monomeric Scap with that of the Scap–Insig dimer (**A**) and cholesterol binding to the AlphaFold model (**B** and **C**). **A** shows the TM region of the Scap–Insig dimer with Scap (blue) and Insig (yellow) aligned to the TM region of the AlphaFold model for Scap (tan). TM helices of Scap are numbered, together with TM helices 3 and 4 of Insig (given the prefix I). The sterol ring system of the digitonin molecule bound at the dimer interface is shown in ball and stick (green). **B** and **C** show views from the cytosolic side of cholesterol binding (green, sticks) to the AlphaFold model, on the cytosolic (**B**) and luminal (**C**) sides of the membrane, respectively

The docking studies described above were performed with Scap and Insig monomers in their dimer conformations. Although no complete high-resolution structure has yet been determined for the Scap monomer, cryo-EM data for the monomer allowed the resolution of TM1 and 3–6 and a density packed against TM4 and 5 that was assigned tentatively to TM8, suggesting distinct differences in TM helix packing for Scap in its monomer and dimer states (Kober et al. 2021). However, a potential new source of structural information is provided by the structure prediction programme AlphaFold (Jumper et al. 2021; Varadi et al. 2022). The structure predicted by AlphaFold for the Scap monomer presumably corresponds to that of the Scap monomer in its non-cholesterol-bound state. AlphaFold was trained on protein structures in the PDB released before 30th April 2018, at which time none of the three currently available Scap–Insig dimer structures (Yan et al. 2021a, b; Kober et al. 2021) had been published. AlphaFold predicts correctly the eight TM helix topology of Scap (Fig. 5A), and the hydrophobic thicknesses predicted by OPM (Lomize et al. 2006) for the lipid bilayers surrounding Scap in the Scap–Insig dimer (PDB: 7ETW) and in the AlphaFold structure are both 28.4 Å. A comparison of the dimer and AlphaFold monomer structures (Fig. 5A) shows that TM helices 1 and 3–6 in the AlphaFold structure align well with those in the dimer structure, consistent with cryo-EM data for the Scap monomer (Kober et al. 2021). In the AlphaFold model, TM2 is further away from the other TM helices than it is in the dimer, possibly explaining why TM2 was not resolved in the cryo-EM structure for the monomer. Similarly, in the AlphaFold model, TM8 adopts a position where it makes no contact with any of the other TM helices and if this same position were to be adopted in the dimer, it could explain why TM8 was not resolved in any of the cryo-EM structures (Yan et al. 2021a, b). However, the most striking observation is that TM7, which in the dimer is packed against TM3, is, in the AlphaFold prediction, packed against TM4 and 5 (Fig. 5A); in the cryo-EM data for the Scap monomer, density in this position was tentatively assigned to TM8 (Kober et al. 2021), but, in the light of the AlphaFold prediction, it is likely to be TM7.

The reliabilities of AlphaFold predictions are given by a per-residue confidence metric called pLDDT, where regions with a pLDDT > 90 are expected to be modelled with high accuracy and regions with a pLDDT between 70 and 90 are expected to correspond to good predictions for backbone structures; predictions for regions with a pLDDT < 70 are of low confidence (Jumper et al. 2021; Varadi et al. 2022). The middle residues for all eight TM helices in the predicted Scap structure have pLDDT values > 70, the average value being 81.2 ± 6.7, suggesting that the locations of these helices are likely to be modelled well, as also suggested by the generally good agreement with the cryo-EM structures. In particular, the middle residue in TM7 has a pLDDT value of 79.9, close to the average value for all the TM helices, suggesting that its predicted position is likely to be valid, despite the large shift in its position from that in the dimer.

**Fig. 6:**
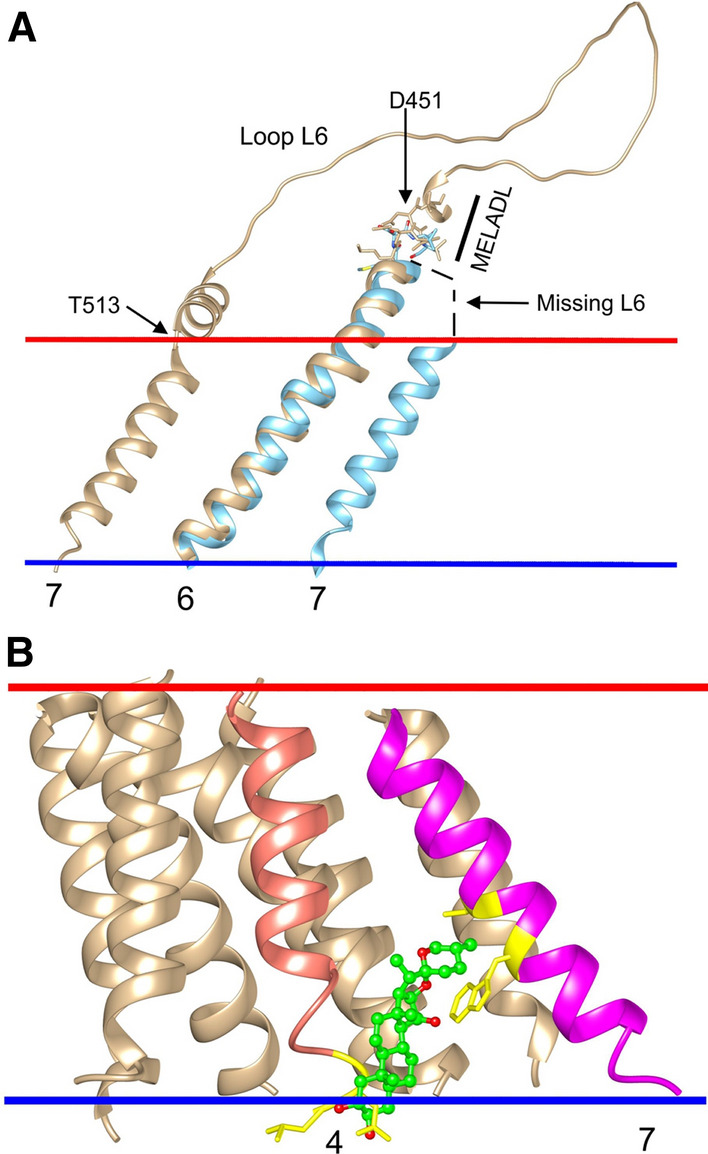
**A** Location of cytosolic loop L6 of Scap in the dimer and monomer structures. TM helices for the AlphaFold model for monomeric Scap (tan) were aligned to those of Scap in the Scap–Insig dimer (blue). The figure shows TM6 and 7 and the connecting loop L6. In the dimer structure, only residues 438 to 450 of L6 are resolved, the missing part being indicated by the broken line. In the AlphaFold model the loop structure between Asp-451 and Thr-513 is judged to be unreliable. The MELADL sequence in L6 is shown in stick format (tan) for residues 447–452 in the AlphaFold model, with the resolved part (residues 447–450) of the sequence in the dimer structure shown in blue. **B** The figure shows why cholesterol does not dock to a dimer-binding site in the AlphaFold model for monomeric Scap. The TM helices are shown for the AlphaFold model for Scap after alignment to the digitonin-bound Scap–Insig dimer; the steroid ring system of digitonin is shown (ball and stick, green); the Scap–Insig dimer itself is not shown. TM4 and TM7 are shown in orange and magenta, respectively; the other helices are coloured tan. Residues in TM4 and TM7 that clash with the digitonin molecule are shown as sticks (yellow)

As expected, the reliability of the predicted structures for long loops between TM helices is less than that for the TM helices. Loop L6, between TM6 and 7, is thought to play an important role in Scap function (Brown et al. 2018); the predicted location of L6 in the Scap monomer structure is shown in Fig. 6A. pLDDT values are high in TM6 up to Ala-450 in the C-terminal extension of TM6 and in TM7 beyond Arg-514 at its N-terminal end, but are low for the intervening loop from Asp-451 to Thr-513, with a value of 30 at Pro-482 in the middle of the loop; the predicted structure for the loop is therefore unreliable, suggesting that the loop could be highly flexible, which would be consistent with the fact that it is not resolved in the cryo-EM structure of the dimer. Within L6, the residues at the C-terminal end of the helix between Met-447 and Leu-452, making up the MELADL motif, are particularly important as they are the binding site for COPII (Brown et al. 2018). The first four residues in this sequence have pLDDT values > 70 although the last two have values of 68.7 and 63.4, respectively; the location of the MELADL motif in the AlphaFold structure is very similar to that part resolved in the cryo-EM structure of the dimer (Fig. 6 A), giving some confidence in the location of these residues. It is also encouraging that the predicted structure for loop L4 matches well the structure for the loop in the Scap–Insig dimer (Fig. 3A of supplementary information), with an average pLDDT value of 84.3 ± 4.5; loop L4 contains two residues, Lys-378 and Arg-380, which are sensitive to proteolytic cleavage and have been used to detect conformation changes in Scap (Gao et al. 2017).

Further differences between the AlphaFold model for monomeric Scap and the structure adopted in the Scap–Insig dimer occur in the region of the dimer-binding site (Figs. 5A, 6B). In the dimer, TM4 is bent so that its N-terminal end will not clash with a cholesterol in the dimer site (Yan et al. 2021a, b) but, in the monomer, TM4 is less bent so that its N-terminal end would now clash with a bound cholesterol (Fig. 6B). Further, if the structure of the monomer were to be unchanged on forming the dimer, TM7 would also partly occupy the dimer site (Fig. 6B). These differences result in differences in the patterns of cholesterol binding between the AlphaFold model of the monomer (Fig. 5B, C) and the Scap monomer in its dimer conformation (Fig. 4C, D). In particular, although binding is observed at the Scap dimer site on monomeric Scap in its dimer conformation (Fig. 4D), no binding is observed in the equivalent region of the AlphaFold model (Fig. 5C); the nearest occupied site for the AlphaFold model is further away from TM4 and, as shown in Fig. 3D, only one of the residues at this site, Ile-348, also contributes to the dimer site (Fig. 3C). Finally, TM7 in the AlphaFold structure would show extensive clashes with TM4 in Insig in the dimer structure; this is avoided in the dimer by moving TM7 to the opposite side of Scap (Fig. 5A).

The fact that the predicted structure for loop L6 is of ‘very low confidence’ (Jumper et al. 2021) suggests that L6 could be highly flexible, and a change in the conformation of loop L6 on formation of the cholesterol-bound dimer is consistent with the observation of changes in the pattern of proteolysis on binding cholesterol (Sun et al. 2007; Brown et al. 2018). In the presence of both cholesterol and Insig, proteolytic cleavage is observed at both Arg-496 and Arg-505 in L6 (Adams et al. 2004; Gao et al. 2017) (Fig. 3 of supplementary information). However, cleavage is observed just at Arg-496 in cholesterol depleted cells in either the absence or presence of Insig, or in the presence of cholesterol but with depleted Insig. Cleavage at Arg-505 in L6 therefore requires both cholesterol and Insig, and so presumable reflects the large change in L6 position occurring on formation of the cholesterol-bound Scap–Insig dimer (Fig. 3 of supplementary information).

Proteolytic cleavage has also been studied for a closely located pair of residues in loop L4 of Scap, Lys-378 and Arg-380 (Fig. 3 of supplementary information) (Gao et al. 2017). In the absence of cholesterol, cleavage was seen at both Lys-378 and Arg-380 in either the absence or presence of Insig, but, in the presence of cholesterol, there was no cleavage at Lys-378 and Arg-380, again in either the absence or presence of Insig. This is surprising since the large conformational changes suggested to be necessary to unblock the MELADL sequence in L6 and allow the binding of COPII, requires the presence of both cholesterol and Insig (Gao et al. 2017) whereas the changes in proteolysis of L4 require only the presence of cholesterol. It is also noticeable that any changes in the structure of L4 between the AlphaFold monomer and the dimer are small, very unlike the large change in structure seen for L6 (Fig. 3 of supplementary information). Further, the surface exposures of Lys-378 and Arg-380 appear to be quite limited (Fig. 3B, C of supplementary information), so that changes in proteolysis could follow from rather general surface changes due to changes in TM packing, rather than from specific changes in L4 itself. It is then possible that the cholesterol binding event to which proteolysis of L4 is sensitive is not related to binding at the dimer site, but could, for example, be binding of cholesterol at one of the binding sites on the TM surface, such as that close to TM4, occupied both in the AlphaFold monomer and dimer structures (Figs. 2C, 5B). However, if the binding event to which proteolysis was sensitive were to be binding at a dimer-binding site on monomeric Scap, then this would require a second conformational state for the Scap monomer, as the AlphaFold structure for the monomer does not contain such a site; this possibility is explored further in the Discussion and Fig. 8.

For Insig, the structure in the dimer is very similar to that predicted by AlphaFold for monomeric Insig (Jumper et al. 2021) and to that of a bacterial homolog of Insig in the absence of steroid (Ren et al. 2015) (Fig. 2 of supplementary information). Cholesterol docking to these structures shows some differences, largely attributable to differences in loop structures on the luminal side, which give rise to small shifts in the predicted positions of the bilayer interfaces around the protein. Importantly, however, all structures show binding at the Insig dimer-binding site (Fig. 2 of supplementary information), that on the AlphaFold model containing 9 of the 11 residues making up the binding site shown in Fig. 3B with a docking energy of – 14.2 kcals mol^−1^, compared to – 14.0 kcals mol^−1^ for the Insig monomer when in its dimer conformation. Other sterols, including 25-HC, also bind to the dimer-binding site on Insig (Table 2 of supplementary information), consistent with mutagenesis experiments that showed the involvement of TM3 and TM4 of Insig in binding 25-HC (Radhakrishnan et al. 2007). There is therefore no suggestion of any major change in the structure of Insig on binding Scap or cholesterol.

### Cholesterol binding to luminal loops L1 and L7

Complex formation between luminal loops L1 and L7 is important for the function of the Scap–Insig complex (Zhang et al. 2016; Gao et al. 2017; Brown et al. 2018). It has been suggested that the loops are separate at high concentrations of cholesterol but bound together at low concentrations (Brown et al. 2018) but cryo-EM studies suggest that the loops are bound together in either the presence or the absence of cholesterol (Kober et al. 2021; Yan et al. 2021a) but with markedly different orientations of the complex relative to the membrane surface (Kober et al. 2021) (Fig. 4 of supplementary information). None of the available cryo-EM structures show cholesterol or other steroids binding in the L1–L7 region (Yan et al. 2021a, b; Kober et al. 2021) but, in direct binding experiments, a construct containing most of loop L1 and a recombinant L1–L7 fusion protein have both been shown to bind cholesterol (Motamed et al. 2011; Zhang et al. 2016). As the locations of these cholesterol-binding sites have not yet been determined, molecular docking is used here to identify potential binding sites for cholesterol on the luminal side of Scap. Since these cholesterol-binding sites are located in loops exposed to water, the docking studies used the default binding parameters established by Trott and Olson (2010) for an aqueous environment.

The validity of the docking approach was first established using a set of structures for membrane proteins with extra-membranous domains containing bound steroids and regions that resemble the L1–L7 complex in Scap (Kober et al. 2021; Yan et al. 2021a) (Table 1 of supplementary information). As an example, Fig. 5 of the supplementary information shows cholesterol-bound and drug-bound states for Niemann–Pick C1-like protein 1 (NPC1L1), with molecules of cholesterol and ezetimibe in a long tunnel connecting the plasma membrane to the N-terminal domain (NTD) where cholesterol could load from a cholesterol-containing micelle (Huang et al. 2020). A docking study was performed on the two structures, covering the whole luminal domain, and a large number of cholesterol poses were observed in the tunnels, overlapping the binding sites for cholesterol and ezetimibe (Fig. 5 of supplementary information). A cholesterol pose was also observed in the NTD, consistent with the suggestion of Huang et al. (2020) that the NTD is the initial cholesterol acceptor (Fig. 5A of supplementary information). Similar results were obtained in docking studies with the other proteins in Table 1 of the supplementary information; the structures show 30 resolved sterols and related molecules, of which 93% were matched by cholesterol poses. Docking studies should therefore be able to detect any binding sites for cholesterol in the luminal domains of Scap.

**Fig. 7:**
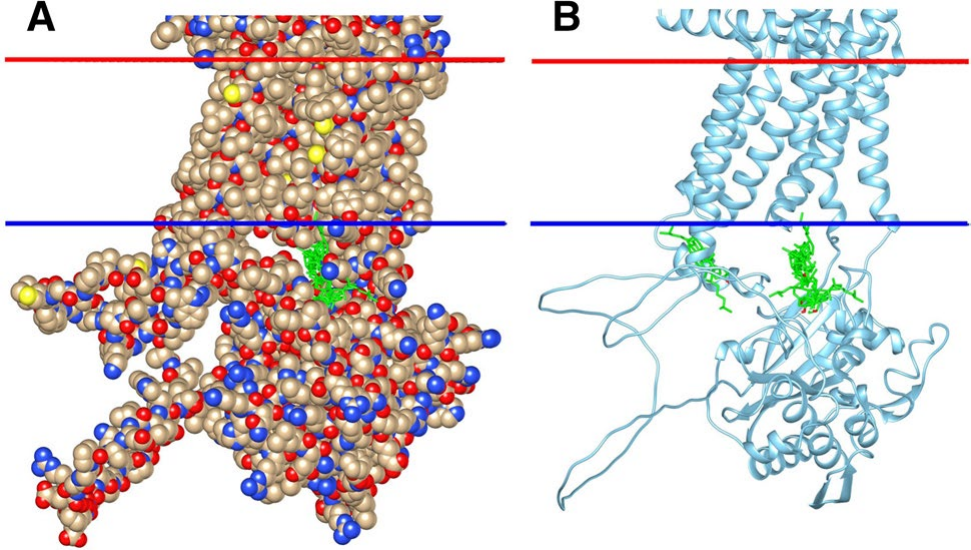
Cholesterol poses on the luminal side of the AlphaFold model for Scap. **A** and **B** show space-fill and ribbon models, respectively, with cholesterol poses in green (sticks). The poses fall into three broad clusters, as shown in Fig. 6 of the supplementary information

Docking to the luminal side of the AlphaFold model for Scap results in three broad clusters of cholesterol poses, the major cluster of six poses being located on one side of a large gap between the L1–L7 complex and the membrane interface, most interactions being with the N-terminal end of L1 (Fig. 7 and Fig. 6 of supplementary information). The very broad range of these poses suggests non-specific interaction with a hydrophobic surface rather than binding at a typical cholesterol-binding site. However, for these sites to be biologically relevant, there must be a mechanism for delivering cholesterol to the sites, as the very low water solubility of cholesterol means that simple collision between a site and a cholesterol free in the aqueous medium will be very rare. In fact, the sites have limited access to the protein surface (Fig. 7) and there is no equivalent to the NTD in NPC1L1 (Fig. 5 of supplementary information) where a cholesterol molecule could be accepted from a loaded micelle or some other carrier. Further, although cholesterol esters are released into the ER lumen for export together with triglycerides, there appears to be no evidence for high concentrations of cholesterol itself in the ER lumen (Morishita et al. 2019; Perkins and Allan 2021). The location of the potential binding sites close to the membrane surface (Fig. 7) might suggest that they could be loaded with cholesterol directly from the membrane, but this would only work if the affinities of the sites on the luminal domain for cholesterol were greater than those on the TM surface of Scap. These affinities can be estimated from docking energies. The average docking energy for the poses on the luminal domain, starting from a cholesterol molecule in an aqueous environment, is – 8.4 ± 0.3 kcal mol^−1^, and the average docking energy for poses on the TM domain, also calculated starting from cholesterol in an aqueous environment, is – 15.8 ± 0.6 kcal mol^−1^. Binding of cholesterol to the TM domain of Scap is therefore favoured over binding to the luminal domain by ca 7.4 kcal mol^−1^, meaning that essentially no cholesterol molecules will move from the membrane to the luminal domain. A similar difference (6.8 kcal mol^−1^) is observed for NPC1L1 (Fig. 5 of supplementary information) but, of course, NPC1L1 moves cholesterol the other way, from the lumen of an enterocyte into the plasma membrane (Huang et al. 2020) so that favourable binding to the membrane is, in this case, essential for function. It seems probable therefore that any binding sites for cholesterol on the luminal domains of Scap will be unoccupied in vivo.

Unfortunately, two key parts of the luminal domain of Scap in the Scap–Insig dimer (PDB: 7ETW) are unresolved, making docking results potentially unreliable in this case, but the poses that were obtained (Fig. 7 of supplementary information) showed average docking energies of 8.1 ± 0.4 kcal mol^−1^ for the luminal domain compared to 16.6 ± 1.0 kcal mol^−1^ for the TM domain, again suggesting that sites on the luminal domain cannot be loaded directly from the membrane and so are unlikely to be occupied in vivo.

### Effects of sterol structure

A wide range of sterols have been shown to cause dimer formation between Scap and Insig in vivo, as shown by their ability to inhibit SREBP cleavage in intact mammalian cells (Radhakrishnan et al. 2007). The sterols were divided by Radhakrishnan et al. (2007) into four classes (Table 2 of supplementary information): class I with an intact steroid nucleus, class II with a hydroxyl or epoxy group on their iso-octyl side chain, class III with a hydroxyl or keto position at the 7 position of the sterol ring, and class IV with modifications to the steroid nucleus. Table 4 of the supplementary information shows the most energetically favourable of the binding poses at the dimer site and Table 2 of the supplementary information compares the functional effects of these sterols with docking energies at the dimer site on the Scap–Insig dimer and on Scap and Insig monomers in their dimer conformations; the structural promiscuity shown in the functional studies is mirrored by the docking studies. Whilst all sterols that retained Scap in the ER membrane showed binding to the Scap–Insig dimer, five of the six sterols that did not result in retention did show binding to the dimer (Table 2 of supplementary information). A possible explanation is that some sterols were unable to access the ER membrane in the in vivo experiments; interactions of sterols with lipid bilayers depend markedly on structure (Atkovska et al. 2018; Kulig et al. 2018) and this could affect levels of partitioning into the membrane and rates of diffusion across the membrane; the ability to mix with phospholipids could also be affected.

## Conclusion

The aim of this paper is to throw light on the interactions between cholesterol, Scap, and Insig in the ER membrane and on how binding of cholesterol results in formation of a Scap–Insig dimer with retention of the dimer in the ER. The molecular docking procedures used in the paper were tested by comparison with the results of cryo-EM and X-ray crystallographic studies. The four digitonin and one 25-HC molecule resolved in the TM region of the Scap–Insig dimer (Yan et al. 2021a, b) were all matched by cholesterol poses (Fig. 2). Cholesterol can also bind to unidentified sites in the extra-membranous, luminal domains of Scap (Motamed et al. 2011; Zhang et al. 2016). Structures of a set of membrane proteins containing domains related to the luminal L1–L7 complex of Scap include 30 resolved sterols and related molecules, of which 93% were matched by cholesterol poses (Table 1 of supplementary information). These comparisons suggest that it is appropriate to use molecular docking to identify sites for cholesterol binding on Scap and Insig and on the Scap–Insig dimer. Of course, the molecular docking approach gives no information about molecular dynamics, but future MD studies based on the docking results (Rosenhouse-Dantsker et al. 2013) could provide such information.

A deep cleft at the interface between Scap and Insig in the dimer (Yan et al. 2021a, b) runs from one side of the membrane to the other (Figs. 1, 2A). The cleft is open to the hydrophobic core of the surrounding lipid bilayer on the cytosolic side, but is only partially open on the luminal side. The luminal side of the cleft provides the dimer-binding site for digitonin and 25-HC (Fig. 1 of supplementary information) (Yan et al. 2021a, b) and is also the location of a cholesterol pose (Fig. 2A, D). The partial closure of the site to the lipid bilayer makes it unlikely that a phospholipid fatty acyl chain will be able to compete with cholesterol for binding at the site, a competition that has been suggested to be common on membrane proteins (Lee 2019b). Cholesterol poses are also observed on the extra-membranous, luminal domains of Scap (Fig. 7 and Figs. 6, 7 of supplementary information) but low concentrations of cholesterol in the ER lumen and the strong preference of cholesterol for binding to the TM region of Scap rather than to these sites, means that they are unlikely to be occupied in vivo. It is suggested therefore that effects of cholesterol on ER retention could follow solely from binding to the dimer-binding site.

A low resolution cryo-EM structure for monomeric Scap suggests that TM helix packing in the monomer differs from that in the dimer (Kober et al. 2021). Comparing the structure predicted for the Scap monomer by AlphaFold (Jumper et al. 2021) with that of the dimer suggests a major change in position for TM7 on formation of the dimer (Figs. 5, 6A) with a concomitant change in loop L6 which connects TM6 and 7; a change in the structure of L6 is consistent with reported changes in the pattern of proteolysis of L6 on binding cholesterol (Sun et al. 2007; Brown et al. 2018) (Fig. 3 of supplementary information). This change in loop structure could explain the retention of Scap in cholesterol-rich ER membranes if it led to burial of the MELADL sequence in the loop, so that it was unable to bind to COPII (Fig. 6A).

Comparing the AlphaFold monomer structure with the digitonin-bound dimer structure identifies a number of structural changes required in the monomer before digitonin could bind and before Scap and Insig could interact to form a dimer (Fig. 6B). In the monomer structure, digitonin would clashes with three residues in TM4 of Scap, Leu-343, Asn-344, and Gln-345 (Fig. 6B) but in the dimer TM4 is bent to prevent such clashes. Also, in the monomer structure, digitonin would clash with two residues in TM7, Thr-524 and Trp-527 (Fig. 6B), prevented in the dimer by the large displacement of TM7 (Fig. 6A). Potential clashes in the monomer structure with a bound cholesterol are very similar to those with digitonin, involving Asn-344, Ile-348 and Phe-349 in TM4 and Ile-520 and Trp-527 in TM7. 

A further important consequence of the displacement of TM7 on forming the dimer is that it avoids all the clashes that would otherwise occur with 19 of the residues in TM4 of Insig (Fig. 5A).

**Fig. 8:**
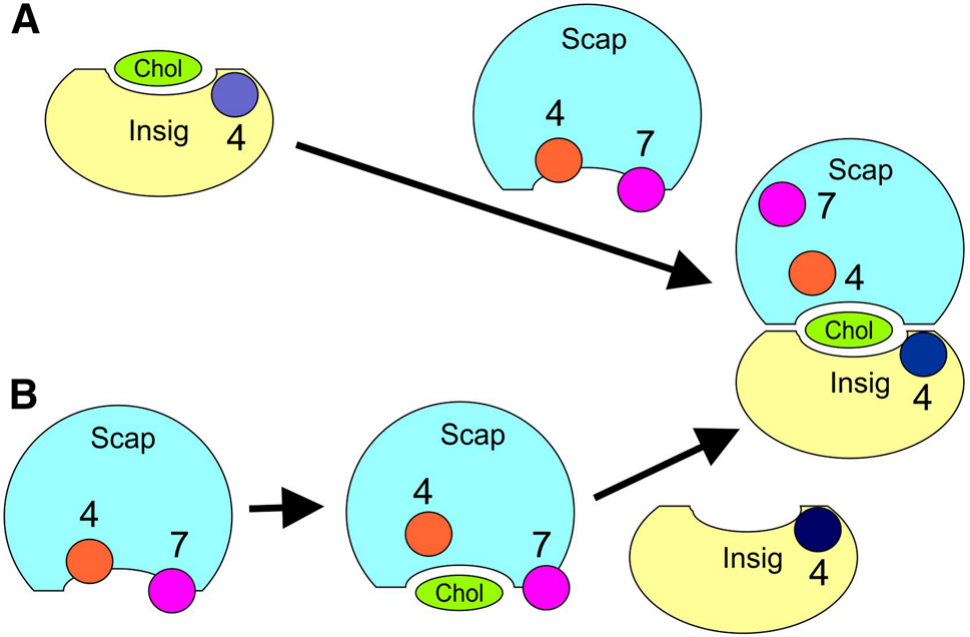
Two possible pathways for Scap–Insig dimer formation and the importance of clashes between TM4 (circle, brown) and TM7 (circle, magenta) in Scap with cholesterol (Chol, green) at the dimer-binding site and between TM7 in Scap with TM4 of Insig (circle, dark blue). In (**A**) the process starts with an Insig molecule (yellow) with cholesterol bound at its dimer-binding site. This collides with a Scap molecule (blue) in which the dimer-binding site is empty because of partial occlusion of the site by TM4 and TM7. Following the collision, TM4 and TM7 undergo small and large displacements, respectively, allowing the Scap–Insig interface to form, resulting in a cholesterol-bound dimer. In (**B**) the process starts with a Scap monomer in which the dimer-binding site is empty because of occlusion of the site by TM4 and TM7. It is proposed that this state could be in equilibrium with a second, intermediate state in which small movements of TM4 and TM7 allow the binding of cholesterol at the dimer-binding site. Collision of the cholesterol-bound, intermediate state with cholesterol-free Insig then leads to the major displacement of TM7 (due to collision with TM4 of Insig), resulting in formation of the cholesterol-bound dimer

Two possible mechanisms for the formation of the cholesterol-bound Scap–Insig dimer are suggested in Fig. 8. The partial closure of the interfacial cleft on the luminal side of the dimer (Fig. 2A) means that direct movement of cholesterol between the lipid bilayer and a pre-formed dimer-binding site is unlikely. Formation of a cholesterol-loaded dimer is therefore likely to start with a collision between a Scap and an Insig molecule, where one of the two already has a cholesterol bound at the dimer-binding site. Cholesterol docking studies with the Insig monomer shows the presence, on the Insig monomer, of a cholesterol-binding site equivalent to the dimer site in the Scap–Insig dimer (Fig. 2 of supplementary information) and, with an association constant of 1.4 × 10^3^ (Table 1), this site will be 98% occupied at a cholesterol content of 5 mol%. Cholesterol docking studies with the AlphaFold model for the Scap monomer suggest that, in this conformation, Scap has no cholesterol-binding site equivalent to the dimer site in the Scap–Insig dimer (Fig. 5C) and so will be present in a cholesterol unbound state. The most likely route to the cholesterol-bound dimer would then be that shown in Fig. 8A, starting with a collision between cholesterol-bound Insig and cholesterol-free Scap. Formation of the dimer would then require movement of TM7 in Scap both to prevent clashes with TM4 in Insig and to prevent clashes with the bound cholesterol, and movement of TM4 in Scap to prevent clashes with the bound cholesterol. Packing of the TM helices at the dimer interface could start with an initial collision between TM7 in Scap and TM4 in Insig, this inducing the movement of TM7 along the unformed Scap–Insig interface to its final position on the opposite side of the dimer, a movement made possible by the long L6 loop. Once TM7 is removed from the interface, the TM helices making up the interface could pack together, in a zipper-like fashion.

Although the AlphaFold model for the Scap monomer has no cholesterol-binding site equivalent to the dimer site, changes in the pattern of proteolysis in loop L4 of Scap have been observed on binding cholesterol in the absence of Insig (Gao et al. 2017). As described above, this could follow from binding to a site not at the dimer interface, or could indicate a second conformation for monomeric Scap, one with a dimer-binding site for cholesterol. Unblocking of the cholesterol-binding site in this second conformation would require a bent TM4 together with a small movement of TM7, sufficient to unblock the cholesterol-binding site but much smaller than the movement seen on binding Insig so that the MELADL sequence remains blocked. This would then give a second pathway to the cholesterol-bound Scap–Insig dimer, with cholesterol binding to the second conformation of **S**cap, followed by binding of unbound Insig, to give the cholesterol-bound dimer (Fig. 8B); this pathway seems less likely as only a small proportion of Insig will be present in the ER membrane in a non-cholesterol-bound state.

As to the nature of the dimer-binding site, docking studies with Scap and Insig in the conformations they adopt in the Scap–Insig dimer show that interactions with Insig make the largest contribution to binding (Table 1, Fig. 3). The –OH group of the bound cholesterol hydrogen bonds to the bilayer interface rather than to the protein (Fig. 3A), as observed for most cholesterol molecules on the TM surfaces of membrane proteins (Lee 2019a, b, 2020, 2021). The sterol ring of digitonin bound at this site occupies the same location as cholesterol, despite the additional two –OH groups and two ring oxygens of digitonin (Fig. 3A), emphasising the structural promiscuity of the binding site which is also shown by the wide variety of sterols that can bind to the site (Table 2 of supplementary information) and inhibit SREBP cleavage in vivo (Radhakrishnan et al. 2007).

## Supplementary Information

Below is the link to the electronic supplementary material.

Supplementary file1 (PDF 1273 KB)
