# Supplementary material for: The role of cholesterol binding in the control of cholesterol by the Scap–Insig system
Source: Eur Biophys J. 2022 Jun 19;51(4-5):385–99. doi: 10.1007/s00249-022-01606-z (PMC9233655; doi:10.1007/s00249-022-01606-z)
Supplement: Supplementary file 1 — Supplementary file1 (PDF 1273 KB) [file 249_2022_1606_MOESM1_ESM.pdf]

Supplementary Information

The role of cholesterol binding in the control of cholesterol by the Scap-Insig system

Anthony G. Lee

A. Estimating binding energies

The binding of cholesterol (C) at a specific binding site on a membrane protein (P) to give the site-cholesterol complex (PC)

$$P + C \leftrightharpoons PC \qquad (1)$$

is defined by an association constant K_a:

$$K_a = c^o[PC]/[P^f][C^f] \qquad (2)$$

or by the corresponding dissociation constant K_d:

$$K_d = [P^f][C^f]/c^o[PC] \qquad (3)$$

where the brackets denote concentrations, the superscript *f* denotes a free, unbound species and c^o is the standard state concentration, usually 1 M or a mole fraction of 1; c^o is usually not included explicitly in the expressions for K_a and K_d (as it has a magnitude of 1) but is implied, resulting in K_a and K_d being dimensionless quantities. In membrane systems, the unit used for concentrations is usually the mole fraction (White et al. 1998). For simplicity, it is usually assumed that cholesterol molecules will be distributed equally between the two monolayers making up the lipid bilayer component of the membrane.

Experiments to measure binding constants are usually performed with a large excess of ligand so that in eqns. 2 and 3, $[C^f] = [C^t]$, where $[C^t]$ is the total concentration of cholesterol. Binding can be characterised by the concentration of cholesterol at which the binding site is 50% occupied, $[C^t]^{50\%}$, and from eqn. 3,

$$[C^t]^{50\%} = K_d \qquad (4)$$

Binding can also be characterised by the standard Gibbs free energy of binding, ΔG^0, which is the value of the Gibbs free energy of binding at the standard state concentration of 1 in units of mole fraction (White et al. 1998): ΔG^0 can be calculated from K_a by:

$$\Delta G^0 = -RT\ln(K_a) = RT\ln(K_d) \qquad (5)$$

where R is the gas constant and T is the absolute temperature, usually taken to be 298°A (25°C). A negative value for ΔG^0 indicates favourable binding of cholesterol.

If binding of cholesterol to a 'site' on the protein is very weak, only a small proportion of the site will be occupied even at very high concentrations of cholesterol and instead of a binding equation, the interaction of cholesterol with the protein can be described as a partition, with the ratio of bound to free cholesterol given by a partition coefficient K_p. In this case the cholesterol is best thought of as not binding to a particular 'site' but as being located in the first solvation shell around the protein (Leonard and Lyman 2021). The partition coefficient K_p can be written as

$$K_p = [C^{first}]/[C^{bulk}] \qquad (6)$$

with a standard Gibbs free energy of partitioning ΔG^0_{part} given by

$$\Delta G^0_{part} = -RT\ln(K_p) \qquad (7)$$

For what follows, it is important to note that ΔG^0_{part} and ΔG^0 differ in that ΔG^0_{part} does not include the $[P^f]$ term.

Autodock Vina predicts the preferred orientation of a cholesterol molecule in a particular site on the protein (the "pose"), and the value of ΔG^0 for that pose; the units used for binding energy are kcals mol^{-1} and the standard state is 1 M, that is, 1 mole of cholesterol per litre of membrane. To convert this to a standard state of a mole fraction of 1, it is assumed that the hydrophobic core of a lipid monolayer is equivalent to liquid hexadecane; 1 litre of hexadecane (molecular weight 226), with a density of 0.77, contains 3.4 moles of hexadecane, giving a conversion factor of -0.73 kcals mol^{-1}. As described in the main text, the calculated ΔG^0 includes both hydrogen bonding of cholesterol to the protein and to the surrounding bilayer interface. Subtracting a value of -6.5 kcals mol^{-1} from ΔG^0 then gives the final value for ΔG^0 for binding at the site, in concentration units of mole fraction.

In Autodock Vina, calculations of docking energies are based on a statistical scoring function (Trott and Olson 2010) and so could be less reliable than those based on molecular dynamic (MD) simulations. Unfortunately, experimental measurements of binding affinities for cholesterol are not available, but it is useful to see how well the results from docking studies compare with those from MD simulations. The most studied of the membrane proteins are the GPCRs and Tables A1 and A2 show a comparison of results for cases where a site identified in MD simulations can be matched with a corresponding docking pose, as given in Lee (2019). Table A1 lists examples where the MD results were given as values of $[C^t]^{50\%}$, from which values for ΔG^0 for binding can be calculated from eqns. 4 and 5. Table A2 lists examples where the MD results were analysed as a partition from which ΔG^0_{part} can be calculated using eqn. 7. This is related to ΔG^0 for binding by

$$\Delta G^0 = \Delta G^0_{part} - RT\ln(1/[P^f])$$

where $[P^f]$ is the concentration of the unbound protein binding site, in mole fraction units. The use of a partition model implies that only a small fraction of the binding sites will be occupied so that $[P^f]$ can be put equal to the total site concentration $[P^t]$; $[P^t]$ is given by $2x$ where x is the molar ratio of protein to lipid used in the MD simulation, and the factor of 2 accounts for the fact that the lipid forms a bilayer. The conversion term $-RT\ln(1/[P^t])$ will usually be large because the mole fraction of protein is small; for example, in the simulations of Lee and Lyman (2012) the molar ratio of lipid/protein was 909, giving a conversion term of -3.6 kcals mol^{-1}.

ΔG^0 values estimated from MD simulations range between -1.5 and -4.7 kcals mol^{-1} except for PDB:3D4S (Table A1) for which ΔG^0 has a value of -12.2 kcals mol^{-1}; it has been suggested that this high value can be attributed to two cholesterol molecules binding in contact with each other on the protein surface (Salari et al. 2018), and so PDB:3D4S will not be included in the following analysis. ΔG^0 values estimated from docking studies range from -3.2 to 4.0 kcals mol^{-1}. The lack of sites with very low ΔG^0 values in the docking studies could follow from the fact that the docking studies were designed to select the most stable of the binding poses, whereas MD simulations presumably contain contributions from a range of poses. The average of all the ΔG^0 values in Tables A1 and A2 are -3.5 ± 1.0 and -3.6 ± 0.3 kcals mol^{-1} for the MD and docking studies, respectively, and suggest close agreement. However, only 5 of the 9 examples show ΔG^0 values differing by less than 0.6 kcals mol^{-1} (RT), all of which correspond to ΔG^0 values estimated from partition coefficients, and, in one case (PDB: 7E2X, Table A1), values differ by a factor of 2.3. Nevertheless, in general, the values obtained by docking do not look unreasonable when compared to those obtained from MD simulations. Further, the relatively small range of values obtained from the docking studies, combined with the fact that all docking poses were chosen to match the characteristics of bound cholesterols observed in X-ray and cryo-EM studies, suggests that

the values could be valuable in comparative studies, even if, in further studies, the absolute values prove to be unreliable.

Table A1 Comparison of docking free energies with free energies of binding derived from the concentrations at which a binding site is 50% occupied in MD simulations.

GPCR	PDB	Site[a]	ΔG^o (kcals mol^{-1})	
			MD simulation	Docking
β2-adrenergic[b]	3D4S	402	-12.2[c]	-4.8[d]
$5HT_{2B}$[b]	4NC3	1203	-4.1	-3.2[d]
μ-opioid[b]	5C1M	404	-2.7	-3.9[d]
$5HT_{1A}$[e]	7E2X	-	-1.5[f]	-3.5[g]

[a] Site as named in PDB file.
[b] Salari et al. 2018.
[c] Unusually high value could be due to a neighbouring bound cholesterol (Salari et al. 2018).
[d] Lee 2019.
[e] Ansell et al. 2021.
[f] Average for ten sites.
[g] Average for 9 sites; data from unpublished study.

Table A2 Comparison of docking free energies with free energies of partition derived from MD simulations.

GPCR	PDB	Site[a]	MD simulation		Docking
			ΔG^0_{part}	ΔG^0	ΔG^0
A_{2A}	5IU4	2402	-0.5[b]	-3.7	-3.9[c]
	5IU4	2402	-1.2[d]	-4.4	-3.9[c]
A_{2A}	2YDO	S1[e]	-0.8[f]	-4.4	-4.0[c]
	2YDO	S3[e]	-1.1[f]	-4.7	-3.3[c]
A_{2A}	3QAK	Averaged[g]	+0.3[h]	-3.3	-3.2[c]
	4EIY	Averaged[g]	+0.5[h]	-3.1	-3.3[c]

[a] Site as named in PDB file.
[b] Corey et al. 2019 using ABFE method.
[c] Lee 2019.
[d] Corey et al. 2019 using WTMetaD method.
[e] Sites as named in Lee and Lyman 2012.
[f] Lee and Lyman 2012.
[g] Averaged over all bound cholesterols.
[h] Leonard and Lyman 2021.

References

Ansell TB, Curran L, Horrell MR, Pipatpolkai T, Letham SC, Song W, Siebold C, Stansfeld PJ, Sansom MSP, Corey RA (2021) Relative affinities of protein–cholesterol interactions from equilibrium molecular dynamics simulations. J Chem Theory Comput 17:6548-6558

Corey RA, Vickery ON, Sansom MSP, Stansfeld PJ (2019) Insights into membrane protein–lipid interactions from free energy calculations. J Chem Theory Comput 15:5727-5736

Lee AG (2019) Interfacial binding sites for cholesterol on G protein-coupled receptors. Biophys J 116:1586-1597

Lee JY, Lyman E (2012) Predictions for cholesterol interaction sites on the A_{2A} adenosine receptor. J Amer Chem Soc 134:16512-16515

Leonard AN, Lyman E (2021) Activation of G-protein-coupled receptors is thermodynamically linked to lipid solvation. Biophys J 120:1777-1787

Salari R, Joseph T, Lohia R, Hénin J, Brannigan G (2018) A streamlined, general approach for computing ligand binding free energies and its application to GPCR-bound cholesterol. J Chem Theory Comput 14:6560-6573

Trott O, Olson AJ (2010) AutoDock Vina: improving the speed and accuracy of docking with a new scoring function, efficient optimization and multithreading. J Comput Chem 31:455-461

White SH, Wimley WC, Ladokhin AS, Hristova K (1998) Protein folding in membranes: Determining energetics of peptide-bilayer interactions. Methods Enzymol, vol 295. Academic Press, pp 62-87

B. A thermodynamic analysis of cholesterol binding to Scap and Insig in the ER membrane.

The concentration of cholesterol in the ER membrane required for half maximal SREBP processing has been estimated to be ca 5.5 mol% at normal levels of Insig and 3.1 mol% when the level of Insig is increased (Radhakrishnan et al. 2008). These values are very different from the cholesterol concentration of 0.0006 mol% required for half occupation of the dimer site on the Scap-Insig dimer, estimated from the association constant in Table 1. However, the following thermodynamic analysis shows that these values are consistent, supporting the suggestion that the effect of cholesterol levels on the processing of SREBP follow from binding at the dimer site on the Scap-Insig dimer.

The following analysis will only consider cholesterol binding at the dimer interface site of the Scap-Insig dimer. It is convenient to consider the process of forming a cholesterol-bound Scap-Insig dimer as consisting of two steps, the first being the binding of Scap (S) and Insig (I) to give a Scap-Insig dimer (SI), defined by an association constant K_1:

$$S + I \leftrightharpoons SI \quad (1)$$

and the second being the binding of cholesterol (C) to the dimer to give the cholesterol-bound dimer (SIC), defined by an association constant K_2:

$$SI + C \leftrightharpoons SIC \quad (2)$$

The overall equilibrium

$$S + I + C \leftrightharpoons SIC \quad (3)$$

can then be defined by the product of the two equilibrium constants, K_1K_2, so that the concentration of the cholesterol-bound dimer is given by:

$$[SIC] = K_1K_2\,[S^f][I^f][C^f] \quad (4)$$

where the square brackets denote concentrations and the superscript *f* denotes a free, unbound species. For simplicity, we will assume that the concentrations of Scap and Insig in the membrane are equal. If these concentrations are denoted [L] then eqn. 4 becomes:

$$[SIC] = K_1K_2\,[L^f]^2[C^f] \quad (5)$$

[It is worth emphasising that a thermodynamic analysis considers just the difference between an *initial* state and a *final* state, and does not consider the *pathway* between the two states; it is not implied that the actual *pathway* followed is the formation of a cholesterol-free dimer followed by binding of cholesterol to the dimer.]

Eqn. 5 shows that the concentration of the cholesterol-bound dimer depends on the concentrations of Scap and Insig in the membrane. These concentrations, expressed in mole fraction units, will inevitably be low because of the high molar ratio of lipid to protein in the membrane: for example, the molar ratio of lipid to protein in the membrane of the sarcoplasmic reticulum is ca 100:1, or 50:1 in each monolayer (Lee 2003). If then, for simplicity, Scap and Insig were of equal concentration in the membrane, and each made up, say, 5% of the protein molecules in the membrane, their mole fractions would be 0.001. Eqn. 5 shows that these low mole fractions will tend to keep low the proportion of the Scap and Insig molecules present as cholesterol-bound dimer. One way to ensure that a large proportion of the Scap and Insig is present as the cholesterol-bound dimer is to make K_1 large, but then the concentration of dimer would be high in the absence of cholesterol, and the system would not work as a probe of cholesterol concentration. The solution is therefore to keep K_1 relatively small but to ensure that the affinity of the dimer for cholesterol (K_2) is high. This can be shown by a simple, model calculation.

Assume, as above, that the mole fractions of Scap and Insig in the membrane are equal, each being 0.001. Scap molecules in the membrane could be present as free Scap, Scap

bound to cholesterol, Scap bound to Insig, or Scap being part of the cholesterol-bound dimer; Insig will be present in the equivalent four forms. For simplicity, we will assume that the proportions of Scap and Insig bound to just cholesterol are low and so can be ignored; binding constants for cholesterol for the Scap and Insig monomers are less than those for the dimer, as suggested by the data in Table 1; it will be shown below that this simplification has little effect on the final result. With this approximation, the free concentrations of Scap and Insig, $[L^f]$, are given by:

$$[L^f] = [L^t] - [SIC] \quad (6)$$

where $[L^t]$ is the total concentration of Scap and Insig,
so that from eqn. 5

$$[SIC] = K_1K_2([L^t]-[SIC])^2[C^f] \quad (7)$$

Now consider the situation where 50% of the Scap and Insig in the membrane are present as cholesterol bound dimer, that is

$$[SIC] = [L^t]/2 \quad (8)$$

Assuming that cholesterol in the membrane is in large excess over Scap and Insig, so that

$$[C^f] = [C^t] \quad (9)$$

and denoting the concentration of cholesterol at which 50% of the Scap and Insig are present as cholesterol-bound dimer as $[C^{0.5}]$, then from eqns. 7-9,

$$[L^t]/2 = K_1K_2([L^t]/2)^2[C^{0.5}] \quad (10)$$

Rearranging eqn. 10 gives:

$$K_1 = 1/(K_2([L^t]/2)[C^{0.5}]) \quad (11)$$

Radhakrishnan et al. (2008) observed half maximal SREBP processing, at normal Insig levels, at a mole fraction of cholesterol of 0.055 (5.5 mol%).
Inserting $K_2 = 1.6 \times 10^5$ (Table 1) into eqn. 11 with a value of $[L^t]$ of 0.001 and a value of $[C^{0.5}]$ of 0.055, gives

$$K_1 = 4.4 \quad (12)$$

This then is the value for K_1 required to give agreement between the estimated affinity of the dimer site on the Scap-Insig dimer for cholesterol (Table 1) and the concentration of cholesterol required for half maximal SREBP processing (Radhakrishnan et al. 2008), assuming that Scap and Insig make up 5 % of the protein molecules in the ER membrane. The actual value for K_1 can only be calculated if the real concentrations of Scap and Insig in the ER membrane are known, which they are not, but these calculations show that, with reasonable values for these concentrations, there will be a value for K_1 that does provide matching. The observation by Radhakrishnan et al. (2008) that the cholesterol concentration required for half maximal SREBP processing decreases with increasing Insig concentration, agrees with the model. The calculated value for K_1 will also be affected slightly by ignoring the possibility that a proportion of the monomeric Scap and Insig could be bound to cholesterol; this assumption will result in a slight over-estimate of the concentrations of free Scap and Insig in eqn. 5 and so to a slight over-estimate of the value of K_1.

Finally, it is important to check that the estimated value for K_1 does not result in a large proportion of Scap and Insig being present as a dimer in the absence of cholesterol, as that would invalidate the use of dimerization as a probe for the level of cholesterol. From eqn. 1, with $K_1 = 4.4$ and Scap and Insig concentrations of 0.001, the concentration of the cholesterol-free dimer [SI] in mole fraction units is 4.3×10^{-6}, corresponding to 0.4% of the total Scap and Insig. This low basal level of dimer allows the use of the Scap-Insig system to detect the cholesterol level in the membrane.

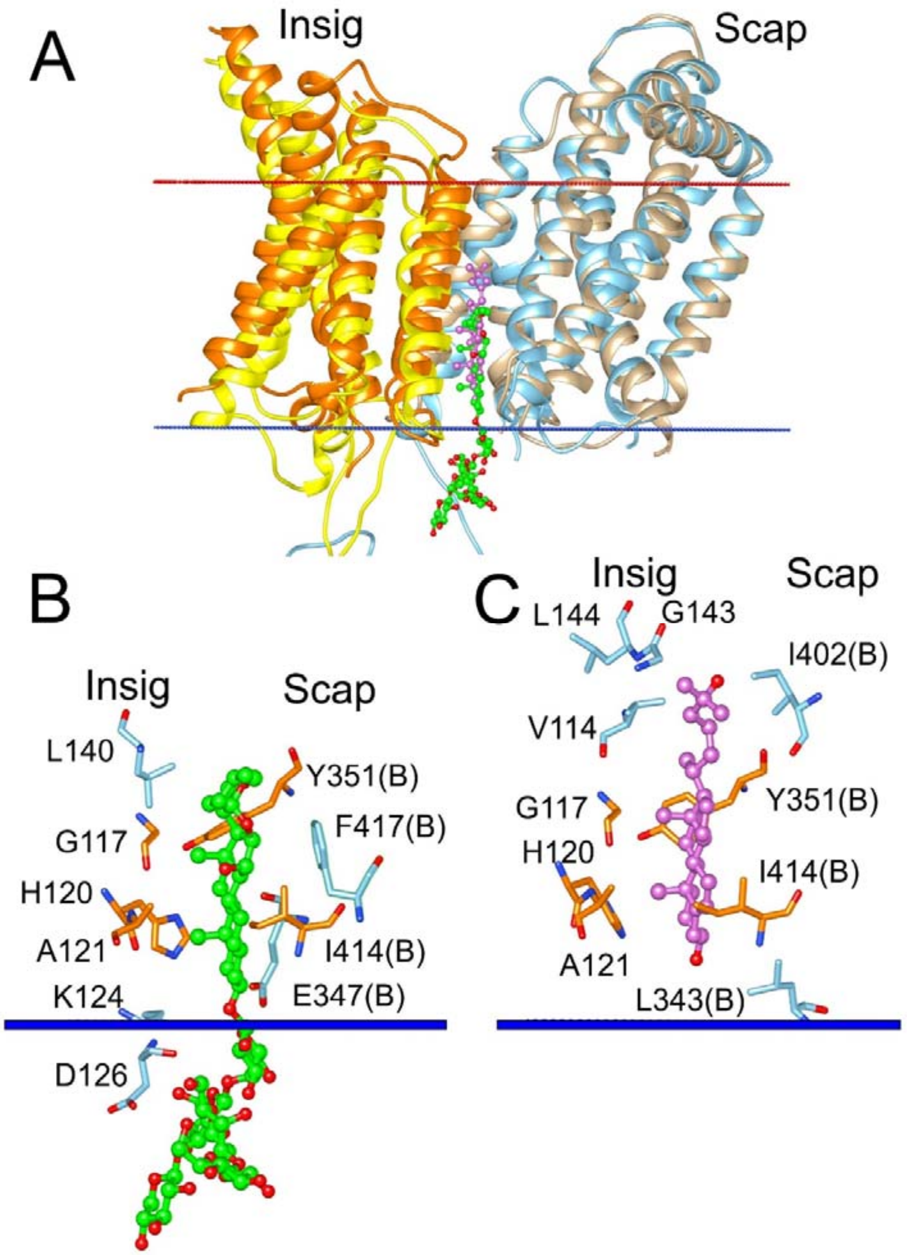

Fig. 1 Steroid binding to the Scap-Insig dimer. (A) shows an alignment of the dimer structure with bound 25-hydroxycholesterol (purple, ball and stick; PDB:6M49) to that with bound digitonin (green, ball and stick; PDB: 7ETW). Insig and Scap subunits are coloured yellow and blue, respectively, for the structure with bound digitonin, and orange and tan, respectively, for the structure with bound 25-hydroxycholesterol. (B) shows residues within 4 Å of the bound digitonin and (C) shows residues within 4 Å of the bound 25-hydroxycholesterol. Residues close to both digitonin and 25-hydroxycholesterol are coloured orange; the other residues are coloured blue.

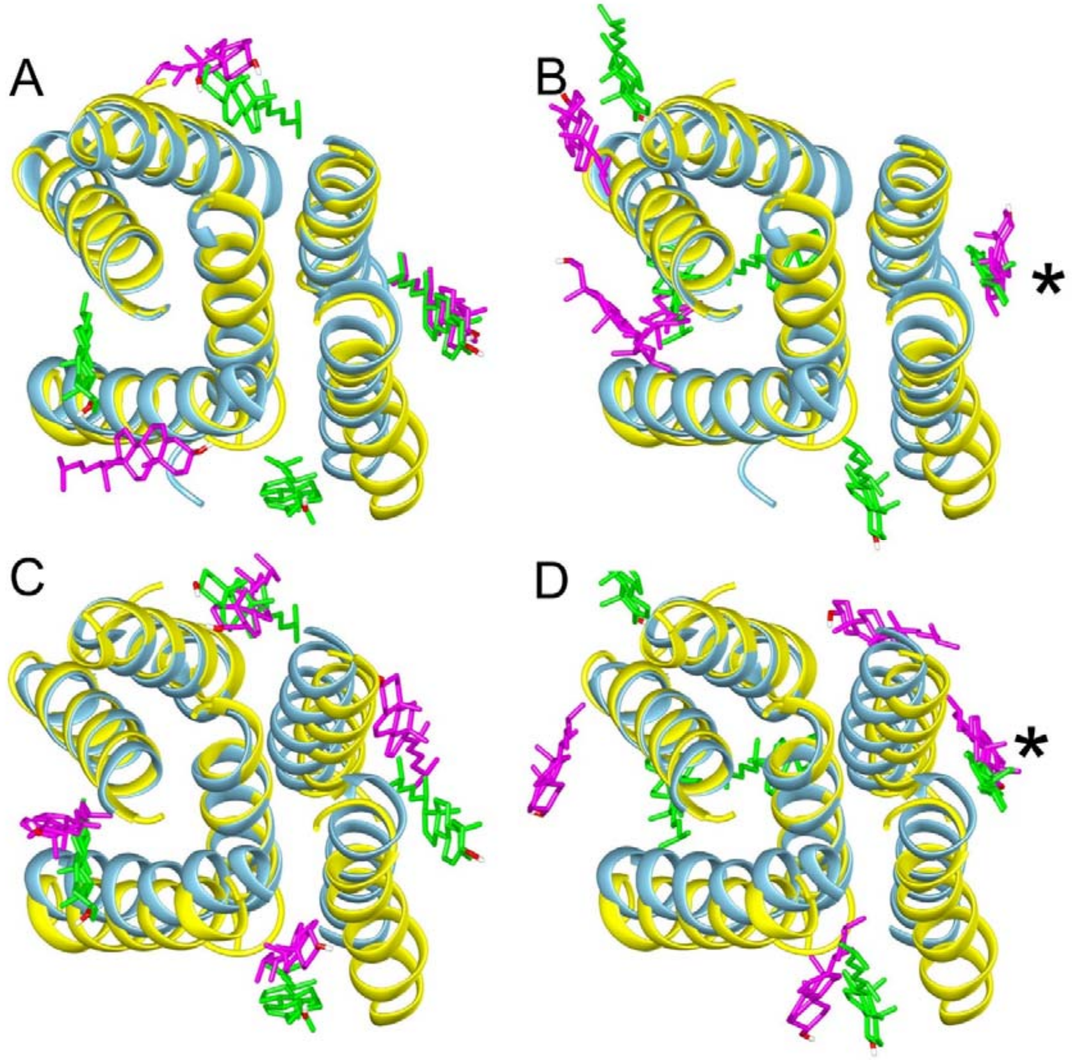

Fig. 2 Cholesterol binding to monomeric Insig. The figure compares binding to monomeric Insig-2 in the structure adopted in the Scap-Insig dimer (yellow) with that for monomeric Insig-2 predicted by AlphaFold (A, B; blue) and with that for bacterial Insig (C, D; blue*)*. All views are from the cytosolic, or equivalent bacterial side, with bound cholesterol shown on the cytosolic (A*,* C*)* and luminal (B*,* D) or equivalent bacterial sides of the membrane, respectively. Cholesterols bound to Insig-2 in the structure adopted in the Scap-Insig dimer are shown in green, and cholesterols bound to the AlphaFold structure or to bacterial Insig are shown in magenta. The asterisks mark poses at the dimer site on Insig. The data for Insig-2 in the Scap-Insig dimer are taken from Fig. 4.

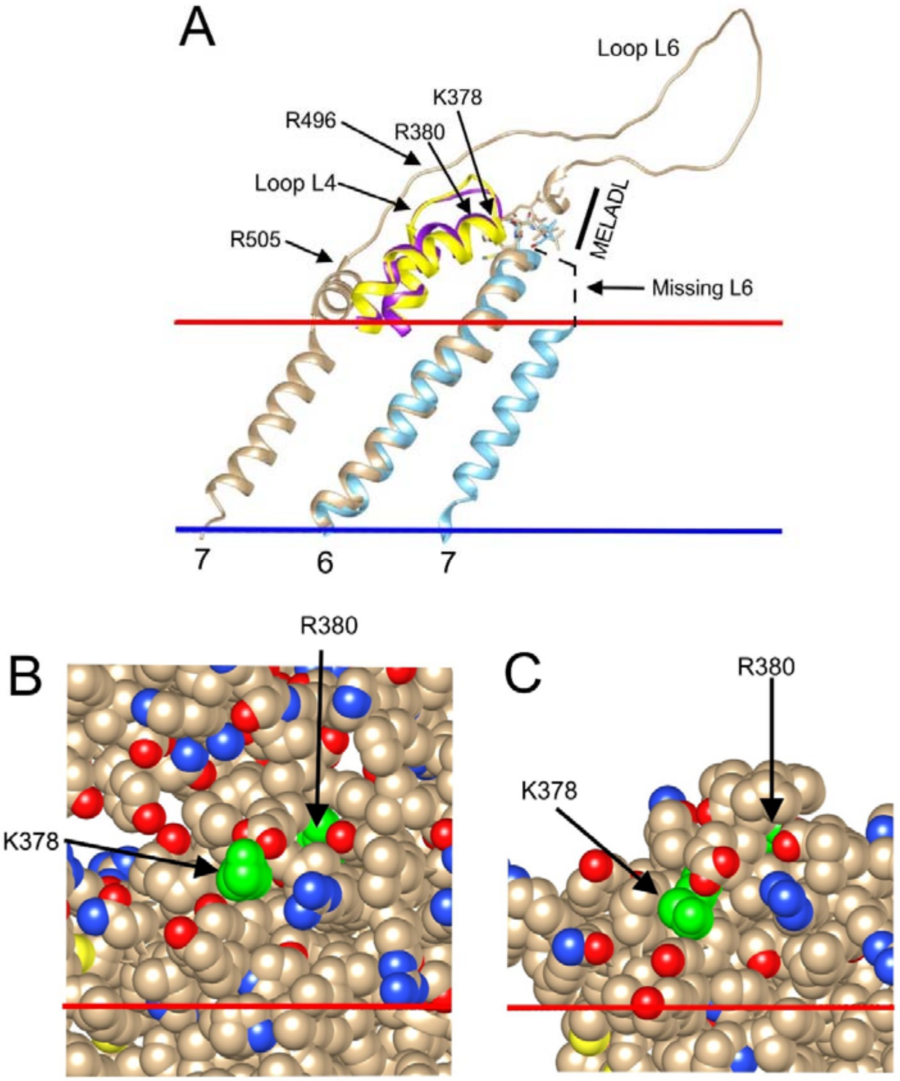

Fig. 3 (A) A comparison of cytosolic loop L4 from Scap in the Scap-Insig dimer (purple) and in the AlphaFold model for the Scap monomer (yellow). TM helices for the AlphaFold model for monomeric Scap (tan) were aligned to those of Scap in the Scap-Insig dimer (blue) as in Fig. 6A. TM helices 6 and 7 are shown, and the proteolytic cleavage sites in L4 at Lys-378 and Arg-380, and in L6 at Arg-496 and Arg-505, are marked. Residues in the MELADL sequence are shown in stick format. (B, C) show surface views, in space fill format, of the AlphaFold model for the Scap monomer (B) and for the Scap-Insig dimer (C) on the cytosolic side; residues Lys-378 and Arg-380 are shown in green. In (C) much of L6 is unresolved, as shown by the broken line in (A).

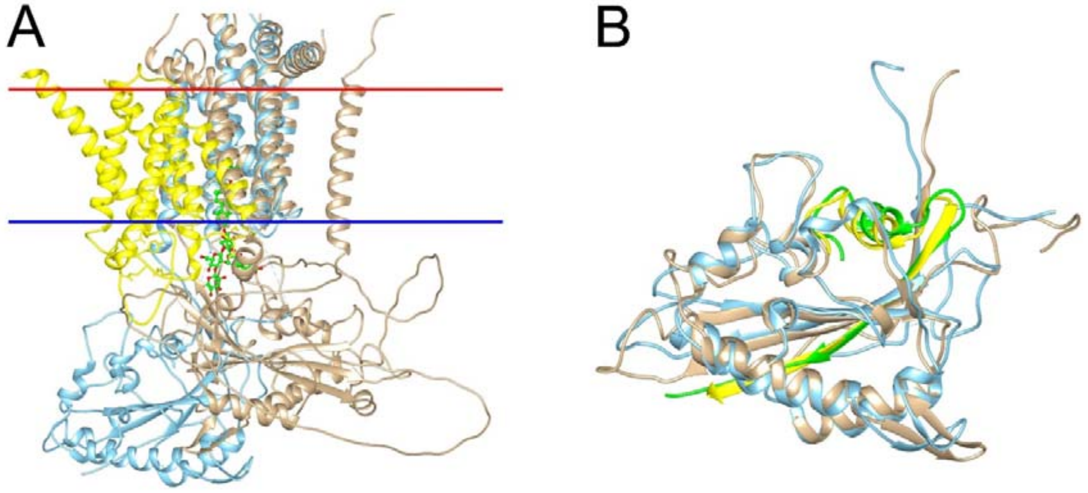

Fig. 4 Luminal loops L1 and L7 in the Scap-Insig dimer and Scap monomer. (A) shows an alignment of the TM helices of the AlphaFold model for Scap (tan) to those of Scap in the Scap-Insig dimer (Scap, blue; Insig, yellow). The digitonin bound at the dimer interface for the Scap-Insig dimer is shown (ball and stick, green). (B) shows an alignment of loops L1 (tan) and L7 (yellow) for the AlphaFold model for Scap to loops L1 (blue) and L7 (green) for the Scap-Insig dimer. Alignment covered the residues resolved in the dimer structure: L1, 69-284; L7, 625-661.

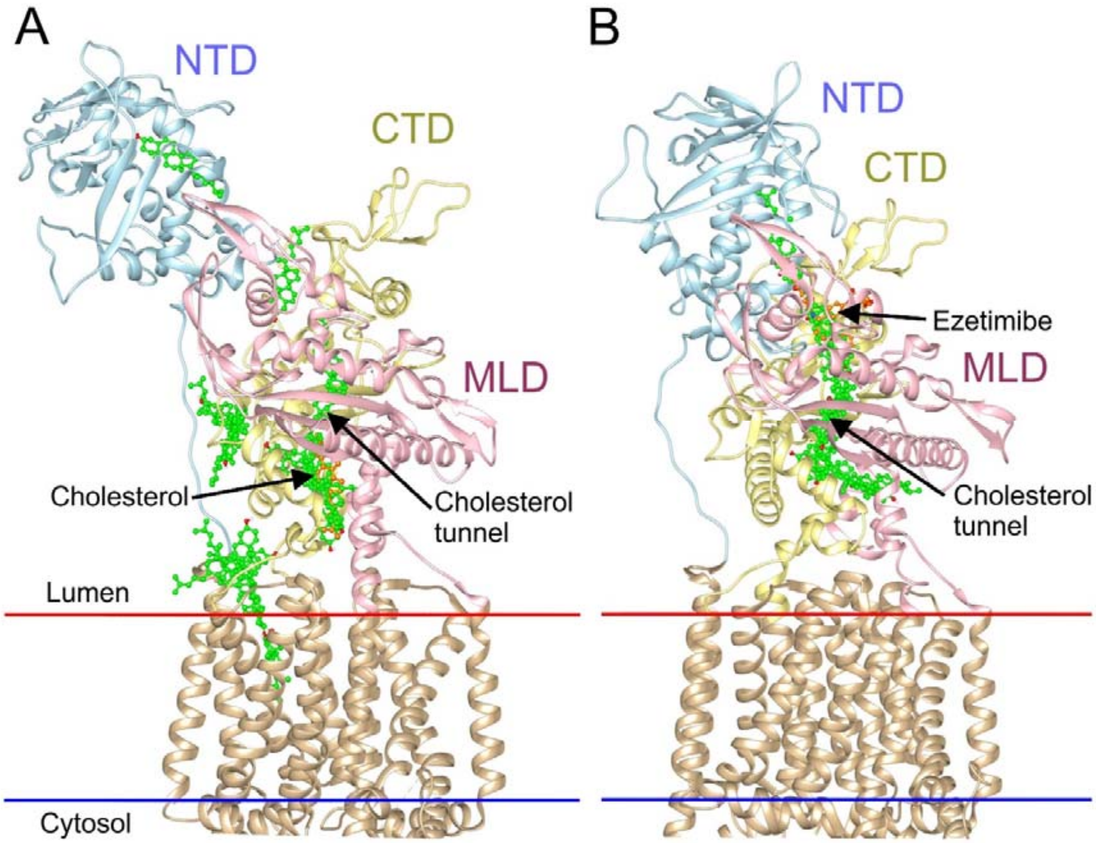

Fig. 5 Cholesterol binding to luminal domains of NPC1L1. The N-terminal domain (NTD), the middle luminal domain (MLD) and the C-terminal domain (CTD) are shown, with a long tunnel located between the MLD and CTD allowing a cholesterol molecule, delivered to the NTD from a cholesterol-containing micelle, to reach the plasma membrane (Huang et al. 2020). (A) shows the structure (PDB: 6V3F) with bound cholesterol (orange, ball and stick) and (B) shows the structure (PDB: 6V3H) with bound ezetimibe (orange, ball and stick). Bound cholesterols are shown in green (sticks).

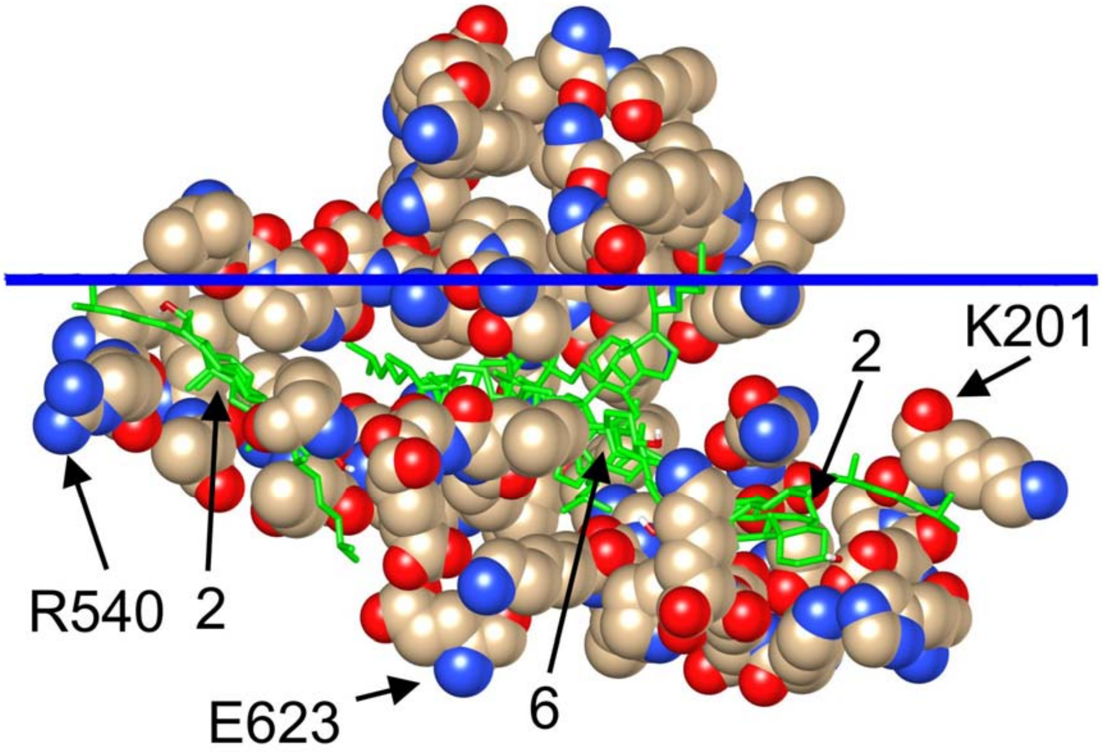

Fig. 6 Residues making up the cholesterol binding sites on the luminal side of the AlphaFold model for Scap. Residues within 4 Å of a cholesterol pose are shown in space fill format. The number of cholesterol poses (green, sticks) in each broad cluster are given. Three residues (K-201, R-540, and E-623) are labelled to allow this view to be oriented with respect to the complete AlphaFold model for Scap.

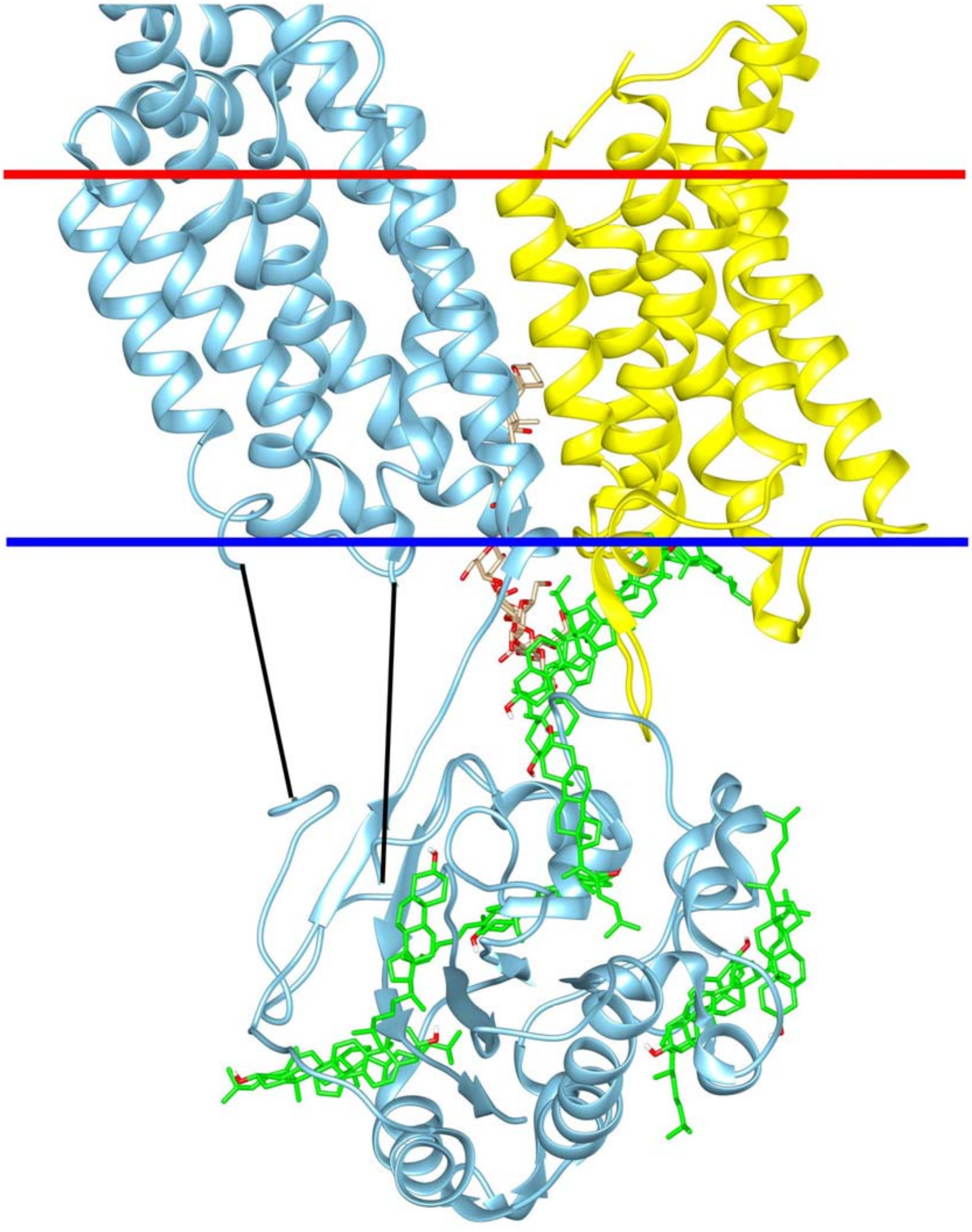

Fig. 7 Cholesterol binding to the luminal side of the Scap-Insig dimer. Scap and Insig are shown in blue and yellow, respectively, with the digitonin bound at the dimer interface shown in tan (sticks). Cholesterol poses are shown in green (sticks). The black lines show where regions of the structure are missing.

Table 1 Matching of resolved sterols and related molecules by cholesterol poses, on proteins containing a sterol-sensing domain (SSD) and an extra-membranous domain related to the L1-L7 complex of Scap.

PDB[a]	Ligand[b]	Matched[c]	Unmatched[c]
NPC1			
3JD8	CLR	A1325	-
6UOX	QDG	A1314	-
6W5R	CLR	A1420	-
6W5S	CLR	A1420	-
6W5T	CLR	A1420, A1421	-
6W5U	CLR	A1420	A1421
6W5V	CLR	A1419,A1420,D204	-
NPC1L1			
6V3F	CLR	A1419	-
6V3H	QO1	A1421	-
7DF8	LMT	A1308	-
7DFW	LMT	A1309	-
7DFZ	QO1	A1306	-
7N4U	VIV	A1407	-
7N4V	CHL	A1405	-
7N4X	CHL	A1405	-
NCR1			
6R4L	ERG	A1201	-
Patched			
6DMB	CHS	A1808	-
6DMY	CHS	A1808	-
6MG8	CHL	A1402	A1401
6N7H	CHL	A1808, A1809	-
6RMG	CHL	A1818	-
6RVD	CHL	B1501	-
7V6Y	CHL	A1201	-
7V6Z	CHL	A1204	-

[a] NPC1, Niemann-Pick Type C protein; NPC1L1, Niemann-Pick C1-like protein 1; NCR1, yeast NPC intracellular sterol transport 1-related protein; Patched, Hedgehog Receptor Patched.

[b] CHS, cholesterol hemisuccinate; CLR, cholesterol; ERG, ergosterol; LMT, dodecyl-β-D-maltoside; QDG, itraconazole; QO1, ezetimibe; VIV, chromanol.

[c] Ligands named as in PDB files.

Table 2 Effects of sterol structure on SREBP cleavage and sterol binding at the dimer binding site

Class	Steroid	Inhibition of SREBP cleavage[a]	Docking energy (kcals mol^{-1})[b]		
			Scap-Insig dimer	Scap[c]	Insig[c]
I	Cholesterol	+	-16.8	-12.5	-14.0
	Dihydrocholesterol	+	-12.9	-10.6	-11.6
	Desmosterol	+	-13.6	-11.2	-11.9
	Androstenol	+	-12.1	nd	nd
	Androstanol	+	-14.8	nd	nd
	25-Fluorocholesterol	+	-13.2	-10.8	-11.6
	Sitosterol	nt	-13.0	-10.5	-11.2
II	25-Hydroxycholesterol	+	-14.4	nd	-12.8
	22-*R*-Hydroxycholesterol	+	-14.7	nd	-12.7
	24-*S*-Hydroxycholesterol	+	-14.5	-11.9	-12.8
	27-Hydroxycholesterol	+	-14.4	nd	-13.6
	24,25-Epoxycholesterol	+	-13.9	nd	-12.6
III	7α-Hydroxycholesterol	+/-	-13.5	nd	-13.0
	7β-Hydroxycholesterol	+/-	-13.8	nd	-12.3
	7-Ketocholesterol	+/-	-12.2	nd	-10.7
IV	19-Hydroxycholesterol	-	nd	nd	nd
	Androstenediol	-	-14.5	nd	nd
	Androstenolone	-	-14.9	nd	-12.4
	Epicholesterol	-	-12.3	nd	-9.0
	4,4-Dimethylcholesterol	-	-14.1	-10.7	-12.1
	Lanosterol	-	-13.2	-11.2	-9.5

[a] Data from Radhakrishnan et al. (2007): nt, not tested (insoluble in MCD); +, maximal effect; +/-, intermediate effect; -, weak effect.

[b] nd, no docking observed.

[c] With the structure adopted in the Scap-Insig dimer.

Table 3 Cholesterol docking to Scap, Insig, and the Scap-Insig dimer

PDB[a]	Chl[b]	E[c]	Local Residues[d]
			Transmembrane Region
			Scap-Insig-2 Dimer
Scap-Insig-2 dimer, human, 7etw			
7etw_CS	C	-14.7	Arg110.A, Val114.A, Gly117.A, Ile118.A, Ala121.A, Leu140.A, Gly143.A, Trp146.A, Thr147.A, Tyr351.B, Val355.B, Thr398.B, Glu399.B, Ile402.B
	D	-14.4	Phe296.B, Ile299.B, Trp313.B, Gly314.B, Ala316.B, Leu317.B, Val320.B, Thr524.B, Trp527.B, Ile528.B
	E	-13.4	Leu133.A, Ser134.A, Ala138.A, Ile142.A, Arg152.A, Phe155.A, Val159.A, Arg189.A, Ser190.A, Pro193.A
	F	-14.3	Thr136.A, Ala139.A, Leu140.A, Ile142.A, Gly143.A, Trp146.A, Ile402.B, Ile405.B, Gly406.B, Ile414.B
	G	-12.7	Ala80.A, Val83.A, Ile84.A, Leu87.A, Ile161.A, Leu164.A, Ala165.A, Val168.A, Thr169.A, Phe196.A
7etw_LS	C	-16.8	Arg110.A, Val114.A, Gly117.A, His120.A, Ala121.A, Lys124.A, Val125.A, Leu140.A, Thr147.A, Phe148.A, Tyr351.B, Val355.B, Ala413.B, Ile414.B, Phe417.B
	D	-15.2	Ser69.A, Trp72.A, Val73.A, Cys76.A, Ala80.A, Ile84.A, Ile161.A, Ala165.A, Val177.A, Phe196.A
	E	-14.6	Phe37.A, Val40.A, Leu44.A, Phe67.A, Ser68.A, Ser69.A, Ala70.A, Trp71.A, Pro74.A
	F	-14.1	Asn130.A, Leu133.A, Ser134.A, Ala138.A, Ile142.A, Phe155.A, Val159.A, Leu186.A, Arg189.A, Ser190.A, Phe197.A
	G	-13.6	Leu285.B, Leu288.B, Val289.B, Tyr292.B, Ile293.B, Phe296.B, Thr534.B, Asp535.B, Gly538.B
	H	-13.4	Ala43.A, Leu46.A, Asn47.A, Leu49.A, Gln50.A, Cys77.A, Ala80.A, Ser81.A, Ile84.A, Phe115.A, Asn119.A, Ser122.A, Ala123.A, Tyr187.A, Leu192.A, Ile195.A, Phe196.A, Gly199.A, Met203.A
	J	-12.8	Leu38.B, Cys41.B, Tyr42.B, Pro43.B, Leu45.B, Lys46.B, Leu519.B, Ala522.B, Val526.B
			Insig (in dimer conformation)
Insig-2 (subunit A) in dimer conformation, human, from 7etw			
7etwA_CS	B	-13.1	Leu133.A, Ser134.A, Ala138.A, Ile142.A, Arg152.A, Phe155.A, Val159.A, Arg189.A, Ser190.A, Pro193.A
	C	-12.5	Val34.A, Ile38.A, Phe41.A, Leu42.A, Val45.A, Trp105.A, Met109.A, Val112.A, Val116.A, Ile206.A, Leu210.A
	D	-12.3	Cys76.A, Val83.A, Leu87.A, Leu157.A, Ile161.A, Leu164.A, Ala165.A, Val168.A
	D	-12.2	Arg110.A, Val114.A, Gly117.A, Ile118.A, Leu140.A, Gly143.A, Trp146.A, Thr147.A
7etwA_LS	B	-15.2	Ser69.A, Trp72.A, Val73.A, Cys76.A, Ala80.A, Ile84.A, Ile161.A, Ala165.A, Val177.A, Phe196.A
	C	-14.6	Phe37.A, Val40.A, Leu44.A, Phe67.A, Ser68.A, Ser69.A, Ala70.A, Trp71.A, Pro74.A
	D	-14.1	Asn130.A, Leu133.A, Ser134.A, Ala138.A, Ile142.A, Phe155.A, Val159.A, Leu186.A, Arg189.A, Ser190.A, Phe197.A
	E	-14.0	Arg110.A, Val114.A, Gly117.A, His120.A, Ala121.A, Lys124.A, Val125.A, Leu140.A, Thr147.A, Phe148.A
	F	-13.4	Ala43.A, Leu46.A, Asn47.A, Leu49.A, Gln50.A, Cys77.A, Ala80.A, Ser81.A, Ile84.A, Phe115.A, Asn119.A, Ser122.A, Ala123.A, Tyr187.A, Leu192.A, Ile195.A, Phe196.A, Gly199.A, Met203.A
			Scap (in dimer conformation)
Scap (subunit B) in dimer conformation, human, from 7etw			
7etwB_CS	het	-14.5	Phe296.B, Ile299.B, Trp313.B, Gly314.B, Ala316.B, Leu317.B, Val320.B, Thr524.B, Trp527.B, Ile528.B
7etwB_LS	A	-13.6	Leu285.B, Leu288.B, Val289.B, Tyr292.B, Ile293.B, Phe296.B, Thr534.B, Asp535.B, Gly538.B
	C	-12.5	Leu343.B, Ile348.B, Tyr351.B, Val355.B, Ile402.B, Ala413.B, Ile414.B, Phe417.B
	D	-12.8	Leu38.B, Cys41.B, Tyr42.B, Pro43.B, Leu45.B, Lys46.B, Leu519.B, Ala522.B, Val526.B
			Bacterial Insig
Insig, bacterial, 4xu4			
4xu4_CS	B	-13.4	Ile21.A, His24.A, Ser25.A, Val28.A, Trp43.A, Ala84.A, Val87.A, Val91.A
	C	-12.8	Thr105.A, Cys109.A, Gly156.A, Leu157.A, Pro162.A, Ala165.A, Pro166.A, Phe169.A
	D	-11.4	Ala84.A, Ala85.A, Gly88.A, Thr92.A, Leu95.A, Val96.A, Phe99.A, Leu107.A
	1	-12.0	Phe12.A, Trp48.A, Ile51.A, Leu52.A, Ala55.A, Leu142.A, Leu145.A, Val147.A

PDB[a]	Chl[b]	E[c]	Local Residues[d]
4xu4_LS	B	-14.6	Arg77.A, Gln78.A, Gly81.A, Gly82.A, Ala85.A, Leu107.A, Ile114.A, Thr115.A, Val118.A, Leu119.A
	C	-12.7	Thr105.A, Trp116.A, Ala125.A, Val129.A, Pro162.A, Ala165.A, Pro166.A, Phe169.A
	D	-13.7	Leu52.A, Ala56.A, Ser59.A, Leu60.A, Leu63.A, Ile131.A, Ile134.A, Gly135.A, Val138.A, Tyr168.A
	E	-12.8	Val10.A, Leu14.A, Ile21.A, Ala79.A, Leu80.A, Val83.A, Leu178.A, Leu181.A, Ala182.A, Arg185.A
			AlphaFold Structures for Scap and Insig
AlphaFold Scap, human			
Scap_CS	B	-14.5	Ile30.A, Ile37.A, Leu38.A, Cys41.A, Leu317.A, Val321.A, Leu328.A, Phe435.A, Val439.A, Ile442.A, Arg446.A
	C	-14.4	Tyr298.A, Tyr351.A, Val355.A, Leu358.A, Glu359.A, Leu362.A, Val507.A, Leu510.A, Ala511.A, Ala516.A
	D	-13.5	Ile348.A, Leu352.A, Val355.A, Leu510.A, Leu515.A, Ala516.A, Leu519.A, Ile520.A
	E	-13.0	Pro287.A, Thr291.A, Ile294.A, Tyr298.A, Tyr351.A, Val506.A, Val507.A, Leu510.A
	F	-12.9	Trp390.A, Met393.A, Lys394.A, Ala397.A, Thr398.A, Gly401.A, Leu404.A, Ile405.A, Phe408.A
	G	-14.4	Ala14.A, Phe15.A, His18.A, Met393.A, Met396.A, Val422.A, Val426.A, Phe429.A, Phe430.A, Met433.A, Leu722.A, Val723.A, Leu726.A
	H	-13.7	His18.A, Leu21.A, Phe32.A, Phe430.A, Leu434.A, Leu722.A, Leu725.A, Leu726.A, Leu729.A, Leu733.A
	I	-13.1	Thr4.A, Leu7.A, Trp390.A, Met393.A, Ala397.A, Leu400.A, Gly401.A, Leu404.A, Val422.A
Scap_LS	B	-13.1	Leu49.A, Pro50.A, Ile280.A, Leu285.A, Leu288.A, Val289.A, Tyr292.A, Phe296.A, Val320.A, Leu324.A, Leu328.A
	C	-13.0	Cys41.A, Leu44.A, Leu45.A, Leu47.A, Leu317.A, Val321.A, Leu324.A, Leu328.A, Phe435.A
	D	-12.4	Asn344.A, Ile348.A, Leu519.A, Ala522.A, Gly523.A, Val526.A, Trp527.A, Ile530.A
	E	-15.1	Met396.A, Leu400.A, Leu419.A, Val422.A, Val426.A, Val711.A, Ala712.A, Gly715.A, Leu716.A
	F	-12.8	Phe32.A, Phe35.A, Cys36.A, Ala39.A, Cys40.A, Tyr42.A, Leu333.A, Leu336.A, Phe430.A, Leu722.A, Leu726.A
AlphaFold Insig-2, human			
Insig_CS	B	-12.4	Leu95.A, Arg152.A, Ser153.A, Gly156.A, Leu157.A, Gly160.A, Phe163.A, Leu164.A, Val167.A
	C	-12.2	Val114.A, Gly117.A, Ile118.A, Ala121.A, Leu140.A, Gly143.A, Trp146.A, Thr147.A
	D	-12.0	Val34.A, Phe37.A, Ile38.A, Val40.A, Phe41.A, Trp105.A, Leu210.A, Tyr213.A
Insig_LS	B	-13.9	Val114.A, Gly117.A, Ile118.A, His120.A, Ala121.A, Lys124.A, Leu140.A, Thr147.A, Phe148.A
	C	-13.9	Trp72.A, Val73.A, Cys76.A, Ala80.A, Val83.A, Ile84.A, Leu87.A, Ile161.A, Leu164.A, Ala165.A, Val168.A, Thr169.A, Leu172.A, Tyr178.A, Phe196.A
	D	-12.0	Trp72.A, Cys76.A, Val83.A, Leu87.A, Ile161.A, Leu164.A, Val168.A, Leu172.A
	E	-12.2	Val29.A, Gly32.A, Val33.A, Phe36.A, Trp71.A, Pro75.A, Thr79.A, Ala82.A, Leu86.A
			Luminal Loops
			Luminal Loops on Scap-Insig-2 dimer
Scap-Insig-2 dimer, human, 7etw			
7etw_LL	C	-9.1	Trp84.B, Ala88.B, Pro89.B, Val90.B, Ala91.B, Leu148.B, Val150.B, Asp152.B, Gln241.B, His242.B, Phe637.B, Tyr640.B, Asn641.B, Ile642.B, Arg647.B
	D	-8.6	Ala111.B, Phe115.B, Ile191.B, Ile192.B, Ile195.B, His196.B, Glu199.B, Thr202.B, Leu203.B, Lys210.B, Asp211.B, Phe214.B, Gly218.B, Lys219.B, Val223.B
	E	-8.4	Lys159.B, Leu160.B, Asn162.B, Leu163.B, Phe177.B, Trp178.B, Gln179.B, Asp181.B, Arg184.B, His198.B
	F	-8.3	Glu80.B, Gln81.B, Gln94.B, Ile96.B, Leu238.B, Ala245.B, Leu248.B, Gly249.B, Leu251.B, Arg252.B, Ala268.B, Glu269.B, Leu652.B, Val654.B
	G	-8.3	Phe97.B, Val98.B, Lys99.B, Val216.B, Pro217.B, Tyr220.B, Ser221.B, Ser233.B, Ser261.B, Pro262.B, Asn263.B, Lys627.B, Leu628.B, Ile655.B, Val657.B
	H	-8.1	Leu148.B, Val150.B, Asp152.B, Leu170.B, Leu172.B, Leu636.B, Phe637.B, Tyr640.B, Ile642.B, Arg647.B
	I	-8.0	Ala88.B, Ala91.B, Leu148.B, Val150.B, Leu170.B, Gln241.B, Leu636.B, Phe637.B, Tyr640.B, Ile642.B, Arg647.B
	J	-8.0	Gln52.A, Ser122.A, Ala123.A, Val125.A, Asp126.A, Phe127.A, Tyr178.A, Gln179.A, Tyr180.A, Asp184.A, Tyr187.A

PDB[a]	Chl[b]	E[c]	Local Residues[d]
	K	-8.0	Asn47.A, Leu48.A, Gln50.A, Ile51.A, Gln52.A, Arg53.A, Asn54.A, Pro60.A, Ser65.A, Ser68.A, Glu279.B, Gly281.B, Arg631.B
	L	-8.0	Glu80.B, Gln81.B, Gln94.B, Ile96.B, Ala245.B, Leu248.B, Gly249.B, Arg252.B, Ala268.B, Glu269.B, Leu652.B, Pro653.B, Val654.B
	M	-7.9	Asn47.A, Leu48.A, Gln50.A, Ile51.A, Gln52.A, Arg53.A, Asn54.A, Pro60.A, Ser65.A, Ser68.A, Glu278.B, Glu279.B, Gly281.B
	N	-7.8	Arg53.A, Asn54.A, Val55.A, Pro59.A, Pro60.A, Val62.A, Ser65.A, Glu278.B, Gly281.B, Val282.B, Ala283.B, Pro634.B, Phe637.B, Ser638.B, Ile642.B, Thr643.B, Leu644.B
	O	-7.8	Phe123.B, Glu127.B, Arg130.B, Asn131.B, Leu153.B, Arg161.B, Asn162.B, Leu164.B, Pro165.B, Glu166.B, His167.B, Cys169.B, Asn176.B, Gln179.B, Asn180.B
	P	-7.7	Arg130.B, Arg161.B, Asn162.B, Pro165.B, Glu166.B, His167.B, Asn176.B, Gln179.B, Asn180.B
			Luminal Loops on AlphaFold Scap
AlphaFold Scap, human			
Scap_LL	B	-9.0	Pro48.A, Pro50.A, Gly51.A, Gly53.A, Pro54.A, Tyr220.A, Thr340.A, Leu343.A, Pro552.A, Leu553.A, Lys627.A, Leu628.A, Ser629.A, Phe630.A
	C	-8.7	Pro48.A, Gly53.A, Pro54.A, Val55.A, Lys277.A, Thr340.A, Leu343.A, Gln550.A, Pro552.A, Leu553.A, Leu628.A, Ser629.A, Phe630.A
	D	-8.5	Leu410.A, Val411.A, Pro412.A, Leu539.A, Arg540.A, Leu543.A, Ala544.A, Val547.A, Thr548.A, Gly554.A, Glu555.A, Ala557.A
	F	-8.4	His196.A, Glu199.A, Pro200.A, Lys201.A, Gln204.A, Ser206.A, Thr208.A, Asp211.A, Pro217.A, Gly218.A, Lys219.A, Lys627.A, Leu628.A, Ser629.A
	G	-8.3	Pro43.A, Lys46.A, Leu47.A, Pro48.A, Pro54.A, Thr205.A, Thr335.A, Leu336.A, Thr340.A, Pro552.A, Leu553.A, Phe630.A
	H	-8.3	Pro48.A, Gly51.A, Gly53.A, Pro54.A, Val55.A, Lys277.A, Thr340.A, Thr342.A, Leu343.A, Asn344.A, Gln550.A, Ser551.A, Pro552.A, Leu553.A, Phe630.A
	I	-8.3	Pro48.A, Gly53.A, Pro54.A, Gln204.A, Asp211.A, Pro217.A, Gly218.A, Lys219.A, Pro552.A, Leu553.A, Lys627.A, Leu628.A, Ser629.A, Phe630.A
	J	-8.1	Leu47.A, Pro48.A, Pro50.A, Gly51.A, Gly53.A, Pro54.A, Val55.A, Lys277.A, Thr340.A, Leu343.A, Gln550.A, Ser551.A, Pro552.A
	L	-8.0	Pro48.A, His196.A, Glu199.A, Gln204.A, Thr205.A, Ser206.A, Thr208.A, Lys210.A, Asp211.A, Pro217.A, Gly218.A, Lys219.A, Lys627.A, Leu628.A, Ser629.A, Phe630.A
	N	-8.0	Leu410.A, Val411.A, Pro412.A, Leu539.A, Arg540.A, Leu543.A, Ala544.A, Val547.A, Thr548.A, Glu555.A, Gly556.A, Ala557.A, Glu623.A

[a] Names of PDB files including bound cholesterols, available for download at https://deepcholesterol.soton.ac.uk. CS, LS, and LL refer to the cytosolic and luminal sides of the membrane, and to luminal loops, respectively.
[b] This gives the letter assigned to cholesterol molecules in the PDB files available for download at https://deepcholesterol.soton.ac.uk.
[c] Docking energies in kcals mol^{-1}.
[d] Local residues within 4 Å of a cholesterol pose: residue numbers are given, together with subunit letter as given in the PDB file.

Table 4 Sterol docking to the dimer site on the Scap-Insig dimer and on Insig and Scap monomers in the configurations they adopt in the dimer.

PDB[a]	E[b]	Local Residues[c]
		PDB files: Scap-Insig dimer (7etw); Insig subunit (7etw_A); Scap subunit (7etw_B)
7-α-Hydroxycholesterol 7AH		
7AH_7etw	-13.5	Arg110.A, Val114.A, Gly117.A, Ile118.A, His120.A, Ala121.A, Lys124.A, Val125.A, Leu140.A, Leu144.A, Phe148.A, Tyr351.B, Val355.B, Ala413.B, Ile414.B, Phe417.B
7AH_7etwA	-13.0	Arg110.A, Ala113.A, Val114.A, Gly117.A, His120.A, Ala121.A, Lys124.A, Val125.A
7- β-Hydroxycholesterol 7BH		
7BH_7etw	-13.8	Val114.A, Gly117.A, Ile118.A, His120.A, Ala121.A, Leu140.A, Leu343.B, Ile348.B, Tyr351.B, Val354.B, Val355.B, Ala413.B, Ile414.B, Phe417.B
7BH_7etwA	-12.3	Arg110.A, Ala113.A, Val114.A, Gly117.A, His120.A, Ala121.A, Lys124.A, Val125.A
7-Ketocholesterol 7KC		
7KH_7etw	-12.2	Val114.A, Gly117.A, Ile118.A, His120.A, Ala121.A, Lys124.A, Val125.A, Leu140.A, Leu343.B, Ile348.B, Tyr351.B, Val354.B, Val355.B, Ala413.B, Ile414.B, Phe417.B
7KH_7etwA	-10.7	Arg110.A, Ala113.A, Val114.A, Gly117.A, His120.A, Ala121.A, Lys124.A, Val125.A
19-Hydroxycholesterol		
[None]		
22R-Hydroxycholesterol 22H		
22H_7etw	-14.7	Val114.A, Gly117.A, Ile118.A, His120.A, Ala121.A, Lys124.A, Leu140.A, Leu343.B, Ile348.B, Tyr351.B, Val355.B, Phe417.B
22H_7etwA	-12.7	Ala113.A, Val114.A, Val116.A, Gly117.A, Ile118.A, His120.A, Ala121.A, Lys124.A, Val125.A, Leu140.A
24,25-Epoxycholesterol 24E		
24E_7etw	-13.9	Arg110.A, Val114.A, Gly117.A, His120.A, Ala121.A, Lys124.A, Val125.A, Leu140.A, Leu144.A, Phe148.A, Tyr351.B, Val355.B, Ala413.B, Ile414.B, Phe417.B
24E_7etwA	-12.6	Arg110.A, Ala113.A, Val114.A, Gly117.A, His120.A, Ala121.A, Lys124.A, Val125.A
24S-Hydroxycholesterol 24H		
24H_7etw	-14.5	Val114.A, Ile118.A, His120.A, Ala121.A, Lys124.A, Val125.A, Leu140.A, Ile348.B, Tyr351.B, Val355.B, Ala413.B, Ile414.B, Phe417.B
24H_7etwA	-12.8	Arg110.A, Ala113.A, Val114.A, Gly117.A, His120.A, Ala121.A, Lys124.A, Val125.A
24H_7etwB	-11.9	Ile402.B, Ile403.B, Ile405.B, Gly406.B, Thr409.B, Val411.B, Ile414.B, Cys418.B
25-Fluorocholesterol FCH		
FCH_7etw	-13.2	Arg110.A, Val114.A, Gly117.A, Ile118.A, His120.A, Ala121.A, Lys124.A, Val125.A, Leu140.A, Leu144.A, Thr147.A, Phe148.A, Tyr351.B, Val355.B, Ala413.B, Ile414.B, Phe417.B
FCH_7etwA	-11.6	Arg110.A, Ala113.A, Val114.A, Gly117.A, His120.A, Ala121.A, Lys124.A, Val125.A
FCH_7etwB	-10.8	Leu343.B, Ile348.B, Tyr351.B, Val355.B, Ile402.B, Ile403.B, Ala413.B, Ile414.B, Phe417.B, Ala421.B
25-Hydroxycholesterol 25H		
25H_7etw	-14.4	Val114.A, Gly117.A, Ile118.A, His120.A, Ala121.A, Lys124.A, Val125.A, Leu140.A, Gly143.A, Leu144.A, Phe148.A, Tyr351.B, Ala413.B, Ile414.B, Phe417.B
25H_7etwA	-12.8	Arg110.A, Ala113.A, Val114.A, Gly117.A, His120.A, Ala121.A, Lys124.A, Val125.A
27-Hydroxycholesterol 27H		
27H_7etw	-14.4	Val114.A, Gly117.A, His120.A, Ala121.A, Lys124.A, Val125.A, Leu140.A, Gly143.A, Leu144.A, Phe148.A, Tyr351.B, Val355.B, Ala413.B, Ile414.B, Phe417.B
27H_7etwA	-13.6	Val114.A, Gly117.A, Ile118.A, His120.A, Ala121.A, Lys124.A, Val125.A, Leu140.A, Gly143.A, Leu144.A, Phe148.A
4,4-Dimethylcholesterol DMC		
DMC_7etw	-14.1	Val114.A, Gly117.A, His120.A, Ala121.A, Lys124.A, Val125.A, Leu140.A, Leu144.A, Phe148.A, Leu343.B, Tyr351.B, Val355.B, Ala413.B, Ile414.B, Phe417.B
DMC_7etwA	-12.1	Arg110.A, Val114.A, Gly117.A, His120.A, Ala121.A, Lys124.A, Val125.A, Leu140.A, Phe148.A
DMC_7etwB	-10.7	Leu343.B, Ile348.B, Tyr351.B, Val355.B, Ile402.B, Ile403.B, Ala413.B, Ile414.B, Phe417.B, Ala421.B
Androstanol ANA		
ANA_7etw	-14.8	Val114.A, Gly117.A, His120.A, Ala121.A, Lys124.A, Val125.A, Tyr351.B, Ala413.B, Ile414.B, Phe417.B

PDB[a]	E[b]	Local Residues[c]
Androstenediol ASD		
ASD_7etw	-14.5	Val114.A, Gly117.A, Ile118.A, His120.A, Ala121.A, Val125.A, Leu343.B, Ile348.B, Tyr351.B, Ala413.B, Ile414.B, Phe417.B
Androstenol ANE		
ANE_7etw	-12.1	Gly117.A, His120.A, Ala121.A, Lys124.A, Val125.A, Leu343.B, Ile348.B, Tyr351.B, Ala413.B, Ile414.B, Phe417.B
Androstenolone ASO		
ASO_7etw	-14.9	Gly117.A, His120.A, Ala121.A, Lys124.A, Val125.A, Tyr351.B, Val355.B, Ala413.B, Ile414.B, Phe417.B
ASO-7etwA	-12.4	Val45.A, Leu48.A, Leu49.A, Gln50.A, Ile51.A, Val116.A, His120.A, Lys124.A
Desmosterol DES		
DES_7etw	-13.6	Arg110.A, Val114.A, Gly117.A, His120.A, Ala121.A, Lys124.A, Val125.A, Leu140.A, Leu144.A, Thr147.A, Phe148.A, Tyr351.B, Val355.B, Ala413.B, Ile414.B, Phe417.B
DES_7etwA	-11.9	Arg110.A, Ala113.A, Val114.A, Gly117.A, His120.A, Ala121.A, Lys124.A, Val125.A
DES_7etwB	-11.2	Leu343.B, Ile348.B, Tyr351.B, Val355.B, Ile402.B, Ile403.B, Ala413.B, Ile414.B, Phe417.B, Ala421.B
Dihydrocholesterol DHC		
DHC_7etw	-12.9	Arg110.A, Val114.A, Gly117.A, Ile118.A, His120.A, Ala121.A, Lys124.A, Val125.A, Leu140.A, Thr147.A, Phe148.A, Tyr351.B, Val355.B, Ala413.B, Ile414.B, Phe417.B
DHC_7etwA	-11.6	Arg110.A, Ala113.A, Val114.A, Gly117.A, His120.A, Ala121.A, Lys124.A, Val125.A
DHC_7etwB	-10.6	Leu343.B, Ile348.B, Tyr351.B, Val355.B, Ile402.B, Ile403.B, Ala413.B, Ile414.B, Phe417.B, Ala421.B
Epicholesterol ECH		
ECH_7etw	-12.3	Val114.A, Gly117.A, Ile118.A, Ala121.A, Lys124.A, Val125.A, Leu140.A, Leu343.B, Ile348.B, Tyr351.B, Val354.B, Val355.B, Ala413.B, Ile414.B, Phe417.B
ECH_7etwA	-9.0	Arg110.A, Ala113.A, Val114.A, Gly117.A, His120.A, Ala121.A, Lys124.A, Val125.A
Lanosterol LAN		
LAN_7etw	-13.2	Gly117.A, His120.A, Ala121.A, Lys124.A, Val125.A, Leu140.A, Leu343.B, Ile348.B, Tyr351.B, Ile402.B, Ile403.B, Ile405.B, Gly406.B, Ala413.B, Ile414.B, Phe417.B
LAN_7etwA	-9.5	Arg110.A, Val114.A, Gly117.A, His120.A, Ala121.A, Lys124.A, Leu140.A, Leu144.A, Phe148.A
LAN_7etwB	-11.2	Leu343.B, Glu347.B, Ile348.B, Tyr351.B, Ile402.B, Ile403.B, Gly406.B, Ile414.B, Phe417.B, Cys418.B
Sitosterol SIT		
SIT_7etw	-13.0	Arg110.A, Val114.A, Gly117.A, His120.A, Ala121.A, Lys124.A, Val125.A, Leu140.A, Gly143.A, Leu144.A, Phe148.A, Tyr351.B, Val355.B, Ala413.B, Ile414.B, Phe417.B
SIT_7etwA	-11.2	Arg110.A, Ala113.A, Val114.A, Gly117.A, His120.A, Ala121.A, Lys124.A, Val125.A
SIT_7etwB	-10.5	Leu343.B, Ile348.B, Tyr351.B, Val355.B, Ile402.B, Ile403.B, Gly406.B, Ala413.B, Ile414.B, Phe417.B

[a] Names of PDB files available for download at https://deepcholesterol.soton.ac.uk. Files are named by the abbreviation for the sterol, followed by: 7etw for Scap-Insig dimer; 7etw_A for the Insig subunit, and 7etw_B for the Scap subunit. Where more than one pose was obtained, the details given in the table correspond to the energetically most favourable of the poses.
[b] Docking energies in kcals mol^{-1}.
[c] Local residues within 4 Å of a cholesterol pose: residue numbers are given, together with subunit letter as given in the PDB file (A, Insig; B, Scap)
